# Autoantibody signature in hepatocellular carcinoma using seromics

**DOI:** 10.1186/s13045-020-00918-x

**Published:** 2020-07-02

**Authors:** Shu Zhang, Yuming Liu, Jing Chen, Hong Shu, Siyun Shen, Yin Li, Xinyuan Lu, Xinyi Cao, Liangqing Dong, Jieyi Shi, Ya Cao, Xiaoying Wang, Jian Zhou, Yinkun Liu, Lei Chen, Jia Fan, Guangyu Ding, Qiang Gao

**Affiliations:** 1 Liver Cancer Institute, Zhongshan Hospital, and Key Laboratory of Carcinogenesis and Cancer Invasion (Ministry of Education), Fudan University, Shanghai, 200032 China; 2 The International Cooperation Laboratory on Signal Transduction, Eastern Hepatobiliary Surgery Hospital, Second Military Medical University, Shanghai, 200438 China; 3 Department of Clinical Laboratory, Cancer Hospital of Guangxi Medical University, Nanning, 530021 China; 4 Department of Thoracic Surgery, Zhongshan Hospital, Fudan University, Shanghai, 200032 China; 5 The Department of Pathology, Eastern Hepatobiliary Surgery Hospital, Second Military Medical University, Shanghai, 200438 China; 6 Institutes of Biomedical Sciences, Fudan University, Shanghai, 200032 China; 7 Key Laboratory of Carcinogenesis and Invasion, Chinese Ministry of Education, Xiangya Hospital and Cancer Research Institute, Xiangya School of Medicine, Central South University, Changsha, 410078 China

**Keywords:** Liver cancer, Early diagnosis, Alpha-fetoprotein, Protein array, Artificial neural network

## Abstract

**Background:**

Alpha-fetoprotein (AFP) is a widely used biomarker for hepatocellular carcinoma (HCC) early detection. However, low sensitivity and false negativity of AFP raise the requirement of more effective early diagnostic approaches for HCC.

**Methods:**

We employed a three-phase strategy to identify serum autoantibody (AAb) signature for HCC early diagnosis using protein array-based approach. A total of 1253 serum samples from HCC, liver cirrhosis, and healthy controls were prospectively collected from three liver cancer centers in China. The Human Proteome Microarray, comprising 21,154 unique proteins, was first applied to identify AAb candidates in discovery phase (*n* = 100) and to further fabricate HCC-focused arrays. Then, an artificial neural network (ANN) model was used to discover AAbs for HCC detection in a test phase (*n* = 576) and a validation phase (*n* = 577), respectively.

**Results:**

Using HCC-focused array, we identified and validated a novel 7-AAb panel containing CIAPIN1, EGFR, MAS1, SLC44A3, ASAH1, UBL7, and ZNF428 for effective HCC detection. The ANN model of this panel showed improvement of sensitivity (61.6–77.7%) compared to AFP (cutoff 400 ng/mL, 28.4–30.7%). Notably, it was able to detect AFP-negative HCC with AUC values of 0.841–0.948. For early-stage HCC (BCLC 0/A) detection, it outperformed AFP (cutoff 400 ng/mL) with approximately 10% increase in AUC.

**Conclusions:**

The 7-AAb panel provides potentially clinical value for non-invasive early detection of HCC, and brings new clues on understanding the immune response against hepatocarcinogenesis.

## Background

Hepatocellular carcinoma (HCC) is one of the leading causes of cancer mortality worldwide [1]. The majority of HCC occur in patients with underlying liver disease, such as hepatitis B virus (HBV) infection and cirrhosis [2]. Over half of patients with HCC are diagnosed at advanced stages, preventing the possibility of curative therapies. Alpha-fetoprotein (AFP) is a widely used, yet imperfect, biomarker for HCC early diagnosis. It has been reported that AFP (at a threshold level of 20 ng/mL) showed low sensitivity of 40–60% with specificity of 80–90% [3]. Low sensitivity, false negativity (e.g., a small HCC with normal AFP level), and false positivity (e.g., liver function damage and certain gastrointestinal tumors) of AFP could lead to decreased chance of early diagnosis and thus poor clinical outcomes, highlighting the requirement for more effective approaches for HCC detection.

Cancer-associated autoantibodies (AAbs) may develop early during carcinogenesis when cancer-associated antigens appear in premalignant or malignant lesions. The immune system can effectively amplify and memorize immune responses to those antigens, thereby making AAbs as appealing cancer biomarkers. For example, DHCR24 AAb was identified as a novel biomarker for disease progression of hepatitis C [4]. Likewise, it has been reported that AAbs against HCC1, CDKN2A, p53, CIP2A, and survivin could indicate the presence of HCC prior to clinical diagnosis [5]. In another study, AAbs against NPM-1, 14-3-3 zeta, and MDM2 were suggested to have diagnostic value for AFP-negative HCC patients (AFP < 20 ng/mL; AFP^−^ HCC) [6]. Serum AAbs against EIF3A [7] and SF3B1 [8] were also reported as potential diagnostic biomarkers for HCC. However, the sensitivity and specificity of those selected AAbs remain limited, and further high-throughput unbiased screening with a large cohort and independent validation are still required. In addition, the heterogeneity of human biology in cancer suggest that combined use of the cancer biomarkers in parallel or in tandem in algorithms such as artificial neural network (ANN) are necessary [9, 10].

Protein microarrays are capable of presenting thousands of tumor-associated antigens to rapidly and globally identify AAb responses in serum (seromics) [11, 12]. Known and predicted tumor antigens have been employed in a comprehensive protein array to profile cancer immune response, such as p53 [13], GPR78 [14], HER2 [15], and HSP60 [16]. In this regard, global AAb screening has identified high-performance AAb panels for early diagnosis of lung cancer [13] and Behcet disease [17]. Herein, the HuProt arrays, comprising of 21,154 unique full-length proteins, were first employed to survey serum AAbs using HCC samples. Subsequently, HCC-focused arrays were fabricated with the candidate proteins identified in the HuProt arrays. A large cohort of 1253 serum samples, including HCC patients, liver cirrhosis (Cirrhotic) patients, and healthy controls (Healthy), were screened to develop a diagnostic model. A novel panel of 7 proteins including CIAPIN1, EGFR, MAS1, SLC44A3, ASAH1, UBL7, and ZNF428 were discovered and evaluated for the early detection of HCC.

## Methods

### Human serum sample

The cohort was comprised of 1253 serum samples from 611 HCC patients, 249 cirrhotic patients, and 393 healthy controls. Between January 2019 and August 2019, these samples were collected at Zhongshan Hospital of Fudan University, Eastern Hepatobiliary Surgery Hospital, and Cancer Hospital of Guangxi Medical University. All blood samples were processed identically to obtain serum. Briefly, 5 mL venous blood was drawn from each individual (before any treatments and surgery), placed in room temperature (RT) for 1 h until coagulated. Serum was recovered by centrifugation at 3000 rpm for 10 min and stored in aliquots at − 80 °C until used. The informed consent and agreement of all samples used in this study have been obtained. The ethical regulations have been approved from each hospital.

Inclusion criteria for HCC patients in this study were (1) pathological diagnosis of HCC (*n* = 446); or (2) diagnosis of HCC by enhanced computed tomography, enhanced magnetic resonance imaging, or contrast-enhanced ultrasonography in combination with AFP or des-gamma carboxyprothrombin for patients without pathological diagnosis (*n* = 165); (3) without autoimmune diseases. Patients were all free of hepatic encephalopathy and ECOG/WHO/Zubrod performance status scored as 0~1. Child-Pugh score, BCLC staging [18], TNM staging, and Chinese Liver Cancer staging [19] were individually estimated; (4) patients with other cancerous history were excluded from our study.

Diagnosis of liver cirrhosis was confirmed by enhanced magnetic resonance imaging or pathology. Healthy controls had normal liver biochemistry and were in the absence of liver diseases and alcohol abuse.

### Serum AAb profiling on HuProt arrays

HuProt™ Human Proteome Microarray v3.0 was provided by CDI Laboratories, Inc (Mayaguez, PR). Each HuProt array is comprised of 21,154 unique proteins. A total of 100 serum samples from discovery phase (I) was applied to HuProt arrays, including 50 HCC and 50 healthy controls. The microarray was taken out from − 80 °C and then incubated in blocking buffer (3% BSA in PBS) at RT for 3 h. Then a serum sample diluted at 1:200 in binding buffer (1% BSA in PBST) was added to the microarray and incubated at 4 °C overnight. After washing with PBST, the microarray was incubated with 1:1000 diluted Fluor conjugated goat anti-human IgG (532 nm) and donkey anti-human IgM (635 nm) (Jackson ImmunoResearch, West Grove, PA) at RT for 1 h in the dark. After washing with PBST, the microarray was rinsed with ddH_2_O and dried. The microarray was scanned with the LuxscanTM 10 K-A (CapitalBio Corporation, Beijing, China). The GenePix Pro 6.0 (Axon Instruments, Foster City, CA) was used for foreground and background intensity extraction for each spot. The signal for each spot (SNR) was defined as the ratio of the foreground to the background median intensity as previously described [20].

### HCC-focused arrays

After serum incubation on the HuProt arrays, autoantibody signals were detected, normalized [21], and quantified. For selection of candidate proteins, three criteria should be satisfied after comparing HCC vs*.* Healthy: (1) *p* values obtained from the *t* test ≤ 0.05; (2) fold change (FC) ≥ 1.2; (3) the positive ratio ≥ 10% (The HCC positive reactivity was defined as greater than the mean plus 2 × SD of the healthy controls. The positive ratio was calculated as the number of HCC positive reactivity to its sum [22]). According to the criteria above, 81 proteins were identified. The extra 19 AAbs including CTRL, DCAF4L2, BIRC5, CCNB1IP1, GPR78, HM13, HSPA2, IMP3, KDM1A, MAPK1, RALA, RPLP0, SARNP, SF3A3, TSPAN13, TUBB6, XRCC5, CENPF, and CDKN2A were selected based on cancer literature in general. We aimed to fabricate the HCC-focused arrays using more candidate proteins from our own experiment and the literature. Thus, a total of 100 proteins were picked to fabricate the HCC-focused arrays, which contained 14 identical subarrays on each slide (BC-BIO, Foshan, China). The subsequent assay process was similar to that described for HuProt array, with an exception that the dilution of serum samples was 1:100 per subarray.

### Model development for HCC detection

For ANN model, we determined the number of hidden neurons based on previous literature [23]. Using the model *N*_h_ = (4n^2^ + 3)/(n^2^ − 8) [*N*_h_, the number of hidden neurons; *n*, the number of input neurons], *N*_h_ was set at 5 in our study. Thus, fully connected feedforward neural-networks including 7 input nodes, 5 neurons in the hidden layer, and 2 output nodes were chosen. Back propagation of error algorithm was used as the learning rule, and the average committee vote was used to classify samples [24–26]. For the test phase (II), 576 samples were randomly split into 10 equally sized groups. One ANN model was constructed using 90% of cases as training set and the remaining 10% as verification set. This procedure was repeated 10 times to obtain 10 ANN models. After repeating 50 times, 500 ANN models were developed. Each ANN model provided the outputs 0 for control or 1 for HCC. The committee vote was performed by averaging all outputs and then to classify the samples. The samples in the validation phase (III) used 500 ANN models for the blind test. Both ANN models and AFP were tested using receiver operating characteristic (ROC) curve analysis.

## Results

### Study design

**Fig. 1:**
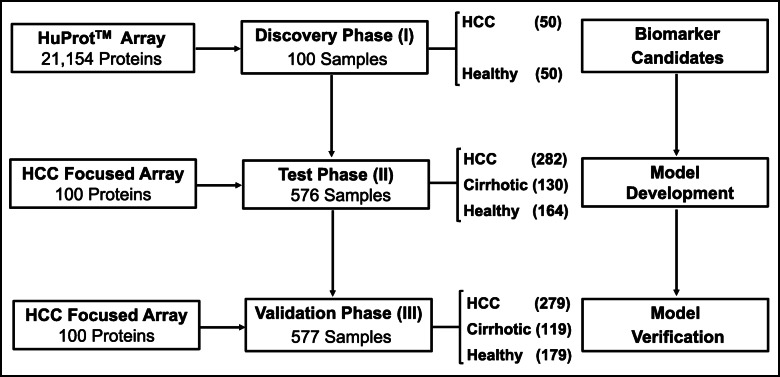
Study design using seromics. A large cohort of 1253 serum samples, including 611 HCC patients, 249 patients with liver cirrhosis (cirrhotic), and 393 healthy controls (healthy), were enrolled for discovery and evaluation of potential serum AAbs as HCC diagnostic biomarkers

This study included three phases (Fig. 1): discovery phase (I), test phase (II), and validation phase (III). In the discovery phase (I), serum samples from 50 HCC and 50 healthy were enrolled. These 100 samples were all obtained from Zhongshan Hospital and individually profiled on HuProt arrays for screening candidate proteins and fabricating the HCC-focused arrays. Then, 282 HCC, 130 cirrhotic, and 164 healthy were collected from Zhongshan Hospital and used for model construction in the test phase (II). Finally, 279 HCC, 119 cirrhotic, and 179 healthy collected from Eastern Hepatobiliary Surgery Hospital and Cancer Hospital of Guangxi Medical University were used for independent verification in the validation phase (III). The clinical data of patients are summarized in Additional file 5: Table S1. Clinical variables of each group in the test phase (II) and validation phase (III) were compared by Pearson’s chi-squared test, and there was no statistical significance.

### AAb screening for construction of HCC-focused arrays

**Fig. 2:**
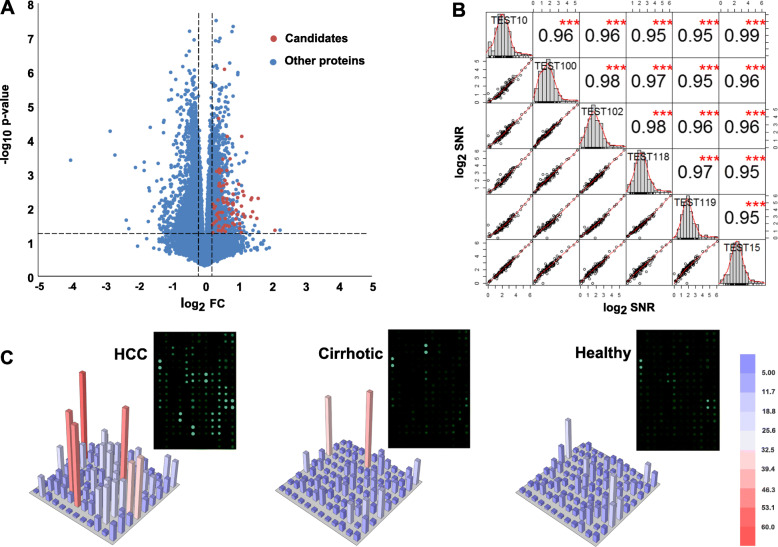
Fabrication of HCC-focused arrays. **a** According to the screening results of HuProt arrays, 81 proteins (*p* ≤ 0.05, FC ≥ 1.2 and positive ratio ≥ 10%) were selected as potential candidates. A total of 100 proteins were printed to fabricate the HCC-focused arrays, including 19 proteins from previous reports. **b** Six representative HCC-focused arrays testing the same sample exhibited high reproducibility. The diagonal indicates the SNR distribution of the sample, the lower left indicates the bivariate scatter plot with a fitted line, and the upper right indicates the correlation coefficient and the significance (***p < 0.001). **c** HCC-focused arrays were incubated with samples from one HCC patient, one patient with liver cirrhosis, and one healthy control, respectively. Three-dimension renderings of the signal intensities were shown, indicating that the array worked well

In the discovery phase (I), the HuProt arrays were employed to profile 100 serum samples collected from 50 HCC and 50 healthy (Additional file 1: Fig. S1). For selection of candidate proteins, three criteria should be satisfied after comparing HCC vs. Healthy, as described in the “Methods” section. Finally, 81 proteins that were more significantly bound by the autoantibodies of HCC group than by those of the healthy group were identified (Fig. 2a). A total of 100 proteins were printed to fabricate the HCC-focused arrays in combination with additional 19 proteins from cancer literature in general. Among these 19 AAbs, 17 were also present in the discovery HuProt array and satisfied 1–2 criteria. Another 2 AAbs, CENPF [16] and CDKN2A [5], were absent in the discovery HuProt array. Alternatively, more samples were enrolled in the test phase (II) and validation phase (III), which would help to accurately evaluate the distinguishing capacity of these AAbs. The biological function and expression level of these 100 proteins were also investigated based on HPA database and our previous multi-omics HCC data [27] (Additional files 2 & 3: Figs. S2 & S3). One serum sample (pooled from 10 randomly selected HCC individuals) was independently applied to a total of 47 different HCC-focused arrays to evaluate their potential variance. As shown in Fig. 2b, the variance was minimal with an average correlation coefficient of 0.95.

### Identification of AAb biomarkers for HCC detection

Next, HCC-focused arrays were tested using serum samples from a large cohort of HCC individually. In the test phase (II), the signals of each protein between HCC and healthy or cirrhotic were compared, respectively. Examples of array image for HCC, cirrhotic, and healthy were provided in Fig. 2c. We identified a total of 55 potential biomarkers using the following criteria: *p* < 0.05, FC ≥ 1.2, and sensitivity > 15% with at least 90% specificity. Among them, 24 AAbs were able to classify HCC patients versus healthy, 17 AAbs were able to classify HCC versus cirrhotic, and the remaining AAbs were able to classify both HCC patients versus healthy and HCC versus cirrhotic (Additional file 6: Table S2).

**Fig. 3:**
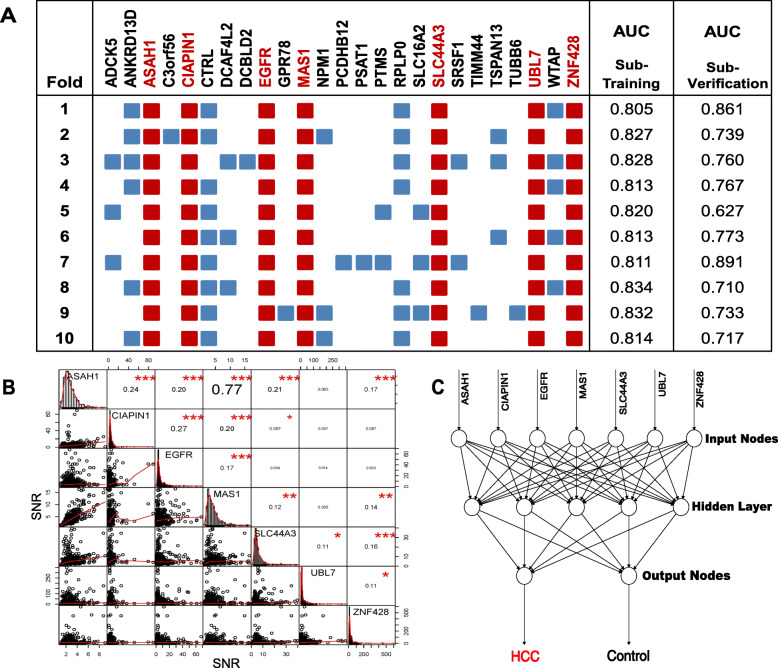
Identification of combinatorial biomarker panel and development of ANN model. **a** Predictors were selected using 10-fold cross validation. The subjects were systematically rotated between ten folds. Within each fold, differential AAbs were determined comparing HCC patients to controls. The predictors for further model development were generated using the potential biomarkers, which worked in ten folds in the cross validation. **b** The correlations between any two proteins from the 7 predictors were calculated using all samples (HCC, cirrhotic, and healthy) in the test phase (II). The diagonal indicates the SNR distribution of the sample, the lower left indicates the bivariate scatter plot with a fitted line, and the upper right indicates the correlation coefficient and the significance (*p < 0.05, **p < 0.01, ***p < 0.001). **c** Schematic representation of the ANN model to predict HCC. Fully connected feedforward neural-networks including 7 input nodes (7 predictors), 5 neurons in the hidden layer, and 2 output nodes were chosen. Back propagation of error algorithm was used as the learning rule, and the average committee vote was used to classify the patient samples

To select predictors for model development, we performed 10-fold cross validation for the 55 potential biomarkers (Fig. 3a). The differential AAbs in each fold were used as input to a logistic regression that classified HCC patients versus controls. Within each fold, stepwise variable selection identified the most discriminative subset of the biomarker candidates [28]. Biomarker candidates selected in ten folds were characterized as predictors in a consensus logistic regression model, and 7 predictors were identified including CIAPIN1, EGFR, MAS1, SLC44A3, ASAH1, UBL7, and ZNF428 (Additional file 4: Fig. S4). The performance of the combinatorial 7 AAbs was then evaluated for HCC detection.

### Performance of the 7-AAb panel in test/validation phase

**Fig. 4:**
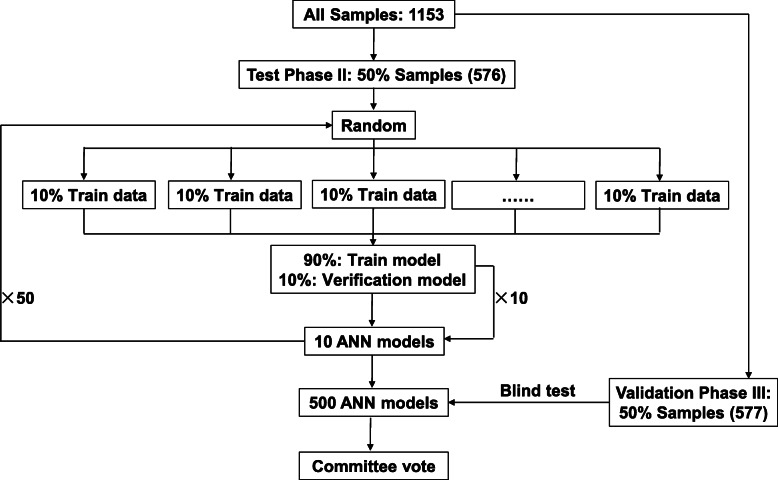
Workflow for the ANN-model. For the test phase (II), 576 samples were randomly split into 10 equally sized groups. One ANN model was built using 90% of cases as training set and the remaining 10% as verification set. This procedure was performed 10 times to generate 10 ANN models. Five hundred ANN models were obtained after a total running of 50 times. Each ANN model provided the following outputs: 0 indicates healthy control and 1 indicates HCC. The committee vote was performed by averaging all outputs and then to classify the samples. The samples in the validation phase (III) used 500 ANN models for the blind test

The correlations between any two proteins from the 7 predictors were calculated using all samples (HCC, cirrhotic, and healthy) in the test phase (II). The results showed that the closest connection existed between MAS1 and ASAH1 with a coefficient of 0.77 (*p* < 0.001; Fig. 3b). It has been reported that neural network analysis was potentially more powerful than traditional statistical techniques when the interaction among variables was complex. Thus, ANN model based on these 7 predictors was further explored in the test phase (II). We built a three-layer neural network with 7 input nodes, 5 hidden neurons, and 2 output neurons (Fig. 3c). The committee vote was performed by averaging all outputs and then to classify the samples (Fig. 4). As shown in Table 1, the ANN model for this 7-AAb panel could identify HCC with a sensitivity of 68.6% and a specificity of 92.1% (AUC = 0.894, HCC vs. controls [healthy + cirrhotic]), which was superior to AFP (cutoff = 400 ng/mL, sensitivity = 28.4%, specificity = 98.7%, AUC = 0.808).

**Table 1:** Performance of the 7-AAb panel and AFP in HCC detection

Phase	Detection^**a**^	HCC vs. (healthy + cirrhotic)			HCC vs. healthy			HCC vs. cirrhotic		
		AUC	Specificity	Sensitivity	AUC	Specificity	Sensitivity	AUC	Specificity	Sensitivity
Test Phase (II)	AFP	0.808	98.7%	28.4%	0.821	100.0%	28.4%	0.789	96.7%	28.4%
	ANN	0.894	92.1%	68.6%	0.933	93.3%	77.5%	0.838	90.2%	61.6%
	AFP + ANN	0.924	92.1%	78.6%	0.959	96.3%	84.1%	0.873	92.4%	71.6%
Validation Phase (III)	AFP	0.822	99.6%	30.7%	0.822	100.0%	30.7%	0.823	98.8%	30.7%
	ANN	0.902	90.1%	73.4%	0.928	93.4%	77.5%	0.853	96.3%	62.2%
	AFP + ANN	0.932	90.1%	82.0%	0.953	93.4%	83.9%	0.893	95.1%	73.0%
Test phase (II) + validation phase (III)	AFP	0.815	99.1%	29.6%	0.821	100.0%	29.6%	0.805	97.7%	29.6%
	ANN	0.898	90.0%	71.6%	0.930	92.7%	77.7%	0.845	90.8%	64.1%
	AFP + ANN	0.928	93.7%	77.0%	0.956	93.4%	85.1%	0.882	91.3%	73.0%

^**a**^The diagnostic cutoff value of AFP was 400 ng/mL

Second, 577 serum samples from an independent cohort were used (phase III) to validate the performance of this 7-AAb panel. Based on the ANN-model, this panel had a sensitivity of 73.4% and a specificity of 90.1% for HCC detection (HCC vs. controls [healthy + cirrhotic], AUC = 0.902) (Table 1), as well as a sensitivity of 80.6% and a specificity of 90.1% for AFP^−^ HCC detection (AFP^−^ HCC vs. Controls [healthy + cirrhotic], AUC = 0.926) (Table 2). Importantly, this panel detected HCC with high sensitivity (62.2–77.5%), outperforming AFP (30.7%) (Table 1).

**Table 2:** Evaluation of the 7-AAb panel in AFP^−^ HCC detection

Phase	AFP^**−**^ HCC vs. (healthy + cirrhotic)			AFP^**−**^ HCC vs. healthy			AFP^**−**^ HCC vs. cirrhotic		
	AUC	Specificity	Sensitivity	AUC	Specificity	Sensitivity	AUC	Specificity	Sensitivity
Test phase (II)	0.898	89.4%	70.4%	0.937	88.9%	83.5%	0.841	87.0%	64.3%
Validation phase (III)	0.926	90.1%	80.6%	0.948	93.4%	83.7%	0.886	85.2%	77.5%
Test phase (II) + validation phase (III)	0.912	89.1%	76.1%	0.942	93.4%	80.3%	0.862	89.6%	65.7%

When combining the test phase (II) and validation phase (III), we found that this model also performed well. It reached to a sensitivity of 71.6% and a specificity of 90.0% in detecting HCC (HCC vs. controls [healthy + cirrhotic], AUC = 0.898) (Table 1), and a sensitivity of 76.1% and a specificity of 89.1% in detecting AFP^−^ HCC (AFP^−^ HCC vs. controls [healthy + cirrhotic], AUC = 0.912) (Table 2).

### The 7-AAb panel’s performance for HBsAg^−^ and HBsAg^+^-HCC

Chronic HBV infection is the leading cause of HCC in Eastern Asian countries and most African countries [3]. Hepatitis B surface antigen (HBsAg) is used to determine whether a patient has a recent or long-standing infection of HBV. In our cohort, approximately 70% (439/611) of HCC patients were HBsAg positive (HBsAg^+^-HCC). For HBsAg^+^-HCC detection, our model provided sensitivity of 59.6–79.1% and specificity of 85.2–95.5%, while AFP (cutoff 400 ng/mL) provided sensitivity of 31.4–34.7% and specificity of 96.7–100% (Additional file 7: Table S3). We also explored the feasibility of the model in HCC patients with negative HBsAg (HBsAg^−^-HCC). Test phase (II) and validation phase (III) contained 46 and 30 HBsAg^−^-HCC patients, respectively. We found that the ANN model of this panel was able to efficiently detect HBsAg^−^-HCC patients from controls (AUC 0.822–0.932), superior to AFP at a cutoff of 400 ng/mL with an AUC of 0.567–0.647 (Additional file 7: Table S3).

### The 7-AAb panel’s performance for different HCC stages

Patients with early-stage HCC can benefit from curative treatments like tumor resection, liver transplantation, or ablation [18]. The performance of our model for HCC patients at different stages were also considered in our study. The evaluation for different stages of BCLC is provided in Table 3 and the others including TNM and Chinese HCC stages in Additional files 8 & 9: Tables S4 & S5. For early stage HCC (BCLC: 0, A; TNM: IA, IB; Chinese: Ia, Ib) detection, our model demonstrated significantly improved performance with 5–20% increases of AUC compared with AFP (cutoff 400 ng/mL). For HCC patients at intermediate or late stages (BCLC: B, C; TNM: II, III, IV; Chinese: II, III) detection, our model in combination with AFP (cutoff 400 ng/mL) achieved sensitivity of 72.2–88.6% and specificity of 89.3–96.6% (AUC 0.887–0.967) for distinguishing HCC patients from controls (healthy and cirrhotic). This combination achieved sensitivity of 79.2–94.3% and specificity of 90.1–97.6% (AUC 0.918–0.985) for distinguishing HCC patients from healthy, and sensitivity of 58.3–88.6% and specificity of 83.7–98.8% (AUC 0.829–0.943) for distinguishing HCC patients from cirrhotic. Thus, the 7-AAb panel based on ANN-model could be effectively applied for early-stage HCC detection.

**Table 3:** Performance of the 7-AAb panel and AFP to detect HCC with different BCLC stages

BCLC stage	Detection^**a**^	Test phase (II)			Validation phase (III)			Test phase (II) + validation phase (III)		
		AFP	ANN	AFP + ANN	AFP	ANN	AFP + ANN	AFP	ANN	AFP + ANN
BCLC (0/A) vs. healthy + cirrhotic	AUC	0.733	0.899	0.906	0.763	0.920	0.924	0.748	0.910	0.915
	Specificity	98.7%	89.9%	92.1%	99.6%	90.1%	90.1%	99.1%	90.0%	89.3%
	Sensitivity	14.3%	69.6%	73.2%	10.5%	80.7%	86.0%	12.4%	72.6%	79.6%
BCLC (0/A) vs. healthy	AUC	0.732	0.937	0.947	0.759	0.942	0.946	0.745	0.940	0.947
	Specificity	100.0%	93.3%	94.1%	100.0%	94.0%	94.7%	100.0%	93.4%	94.4%
	Sensitivity	14.3%	76.8%	82.1%	10.5%	84.2%	86.0%	12.4%	80.5%	84.1%
BCLC (0/A) vs. cirrhotic	AUC	0.736	0.844	0.846	0.772	0.880	0.884	0.753	0.860	0.862
	Specificity	96.7%	97.8%	94.6%	98.8%	91.4%	91.4%	97.7%	90.8%	91.3%
	Sensitivity	14.3%	55.4%	64.3%	10.5%	70.2%	75.4%	12.4%	65.5%	67.3%
BCLC (B) vs. healthy + cirrhotic	AUC	0.824	0.894	0.923	0.847	0.895	0.927	0.835	0.895	0.926
	Specificity	98.7%	89.4%	90.7%	99.6%	90.1%	90.1%	99.1%	90.0%	89.3%
	Sensitivity	28.6%	72.8%	80.3%	33.0%	69.6%	78.3%	30.5%	71.4%	81.3%
BCLC (B) vs. healthy	AUC	0.842	0.933	0.958	0.850	0.922	0.950	0.845	0.928	0.954
	Specificity	100.0%	93.3%	96.3%	100.0%	91.4%	91.4%	100.0%	93.4%	93.4%
	Sensitivity	28.6%	79.6%	85.0%	33.0%	78.3%	85.2%	30.5%	77.1%	85.1%
BCLC (B) vs. cirrhotic	AUC	0.797	0.838	0.871	0.841	0.843	0.885	0.817	0.842	0.879
	Specificity	96.7%	90.2%	94.6%	98.8%	88.9%	88.9%	97.7%	91.3%	91.3%
	Sensitivity	28.6%	61.2%	68.7%	33.0%	65.2%	73.9%	30.5%	62.2%	72.5%
BCLC(C) vs. healthy + cirrhotic	AUC	0.878	0.924	0.962	0.915	0.898	0.959	0.899	0.909	0.959
	Specificity	98.7%	92.5%	93.4%	99.6%	96.6%	96.6%	99.1%	95.0%	94.3%
	Sensitivity	42.9%	74.3%	88.6%	52.1%	70.8%	83.3%	48.2%	71.1%	85.5%
BCLC (C) vs. healthy	AUC	0.899	0.956	0.985	0.923	0.923	0.974	0.912	0.938	0.979
	Specificity	100.0%	92.6%	93.3%	100.0%	93.4%	94.7%	100.0%	93.0%	93.4%
	Sensitivity	42.9%	82.9%	94.3%	52.1%	77.1%	87.5%	48.2%	79.5%	90.4%
BCLC (C) vs. cirrhotic	AUC	0.848	0.877	0.928	0.899	0.850	0.931	0.877	0.861	0.927
	Specificity	96.7%	92.4%	92.4%	98.8%	85.2%	85.2%	97.7%	90.2%	93.1%
	Sensitivity	42.9%	65.7%	85.7%	52.1%	75.0%	87.5%	48.2%	71.1%	80.7%

^**a**^The diagnostic cutoff value of AFP was 400 ng/mL

## Discussion

Although pathological and radiological examination remains the “gold standard” for clinical diagnosis of cancers, liquid biopsy has shown appealing potential for early detection of HCC [29]. In this regard, tremendous efforts have been made on the early diagnostic potential of circulating micro-RNA signature [30], cell-free DNA [31], metabolites [32], glycans [33], and DNA methylation pattern [34]. However, AFP is still the only widely used clinical protein biomarker for HCC diagnosis, although approximately 40% of HCC cases harbored a normal AFP level. Due to the nature of stability and easy detection, efforts have also been made to evaluate novel protein biomarkers for HCC detection, such as Dickkopf-1 [35] and Aldo-keto reductase family 1 member B10 [36].

Based on three steps for biomarker classifier development [37], we focused on CIAPIN1, EGFR, MAS1, SLC44A3, ASAH1, UBL7, and ZNF428, which are mainly involved in activation of signaling cascades and apoptotic/metabolic processes. The molecular function of UBL7 is polyubiquitin modification-dependent protein binding, and loss of ubiquitin-proteasome players were suggested to lead to protein expression alteration and hepatocarcinogenesis [27]. CIAPIN1 was reported to play an important role in HCC proliferation through regulating the expression of cell cycle-related proteins [38]. EGFR is a transmembrane receptor tyrosine kinase and plays a key role in HCC development and progression [39]. The biological functions of MAS1, SLC44A3, ASAH1, and ZNF428 in HCC were rarely reported. Here, we provided autoantibody clues for further exploring their biological significance in HCC.

It has been reported that neural network analysis was potentially more useful than traditional statistical techniques when the relationship among variables was complex and non-linear [10]. The performance of ANN-based 7-AAb model could be further improved due to continuous learning of neural networks in future clinical application. However, there are several limitations in the present study. First, AAbs were reported to appear in multiple cancer types due to immune surveillance. Alternatively, it may indicate the potential of AAbs for monitoring various cancer types, similar to the pan-cancer diagnostic value of cfDNA alterations [40]. Based on previous literature, AAb against ASAH1 could be applied to monitor the progression of melanoma [41]. However, there were no significant differences in AAb against EGFR between patients with breast cancer and controls [42]. Thus, further analyses are required to evaluate the diagnostic value of our 7-AAb panel in diverse cancers. Second, this study was conducted using most of patients with HBV-related HCC from China and HCC patients with high ANN value featured HBsAg positivity (*p* value < 0.001, Pearson’s chi-squared test). A prospective multi-nation validation is necessary for further application. Third, the panel contained 7 biomarkers for ANN-model and it is more complex than single marker detection in clinic. Albeit its complexity, ANN could perform better when subclasses are separated by a non-linear boundary.

## Conclusions

In summary, a comprehensive seromic survey was performed for discovering and validating serum diagnostic biomarkers in HCC. Based on ANN-model, we identified a 7-AAb panel that was generally superior to AFP for HCC detection, and performed well for AFP-negative HCC and HCC at early stage. The 7-AAb panel provides potentially clinical value for non-invasive early detection of HCC, and brings new clues on understanding the immune response against hepatocarcinogenesis.

## Supplementary information

**Additional file 1:.** Fig. S1. Boxplot of signals obtained from HuProt^TM^ array. In discovery phase (I), boxplot of signal intensities (SNR = median foreground intensity/median background intensity) was shown after normalization. The red boxes represent 50 HCC samples and the other boxes represent 50 healthy controls. The antigens were recognized by human autoantibodies of the IgG (A) and IgM (B) isotypes, respectively.

**Additional file 2:.** Fig. S2. One hundred proteins for HCC Focused Arrays. (A) A total of 100 proteins were selected to prepare HCC Focused Arrays using GST fusion proteins. Anti-GST antibody was employed for quality control of HCC Focused Array. (B) Two spots (spot 1 and spot 2) of each protein were printed onto array with correlation coefficient (R^2^) approximate of 0.99. (C) Function enrichment analysis identified biological processes, including focal adhesion, and negative regulation of cellular component organization and protein modification process.

**Additional file 3: **Fig. S3. Expression level for the 100 proteins. (A) Gene expression level of 100 proteins in the liver compared to other tissues using The Human Protein Atlas (http://www.proteinatlas.org/). Based on this database, 7 proteins were absent in the liver, including MAS1, C3orf56, DCAF4L2, DEFB112, GPR78, PAGE1 and SCGB1C2. 16 genes showed increased mRNA expression level in the liver. Liver elevated proteins reported in the liver-specific proteome of The Human Protein Atlas were labeled with green. (B) Totally, 64 of these 100 proteins were found and further analyzed in paired HCC tumor and adjacent non-tumor liver tissues according to our previous proteomics (Ref. *Cell*. 179, 561-577 (2019)). (C) Among the 64 proteins, 10 were down-regulated and 8 were up-regulated significantly in HCC tumor, compared with adjacent liver tissues (adjusted *p* < 0.05 and∣log_2_ FC∣ > 0.5) according to our previous proteomics (Ref. *Cell.* 179, 561-577 (2019)). ( 4412 kb)

**Additional file 4:.** Fig. S4. The representative blots for both HCC and controls. (A) HCC Focused Arrays incubated with HCC, liver cirrhosis (Cirrhotic), and healthy control (Healthy), respectively. (B) Performance of CIAPIN1, EGFR, MAS1, SLC44A3, ASAH1, UBL7 and ZNF428 in the test phase (II).

**Additional file 5:.** Table S1. Summary of the study subjects.

**Additional file 6:.** Table S2. Performance of 55 potential biomarkers in the test phase (II).

**Additional file 7:.** Table S3. Performance of the 7-AAb panel and AFP in HBsAg^+^/HBsAg^-^-HCC detection.

**Additional file 8:.** Table S4. Performance of the 7-AAb panel and AFP to detect HCC with different TNM stages.

**Additional file 9:.** Table S5. Performance of the 7-AAb panel and AFP to detect HCC with different Chinese HCC stages.

## Data Availability

The datasets used and/or analyzed during the current study are available from the corresponding author on reasonable request.

## Abbreviations

HCC: Hepatocellular carcinoma
AFP: Alpha-fetoprotein
AFP^−^HCC: AFP-negative HCC
ANN: Artificial neural network
AAb: Autoantibody
HBV: Hepatitis B virus
Cirrhotic: Liver cirrhosis
Healthy: Healthy control
FC: Fold change
ROC: Receiver operating characteristic
AUC: Area under ROC curves
