# Supplementary figures and images for: Autoantibody signature in hepatocellular carcinoma using seromics
Source: J Hematol Oncol. 2020 Jul 2;13:85. doi: 10.1186/s13045-020-00918-x (PMC7330948; doi:10.1186/s13045-020-00918-x)

**Supplementary Fig. S1**

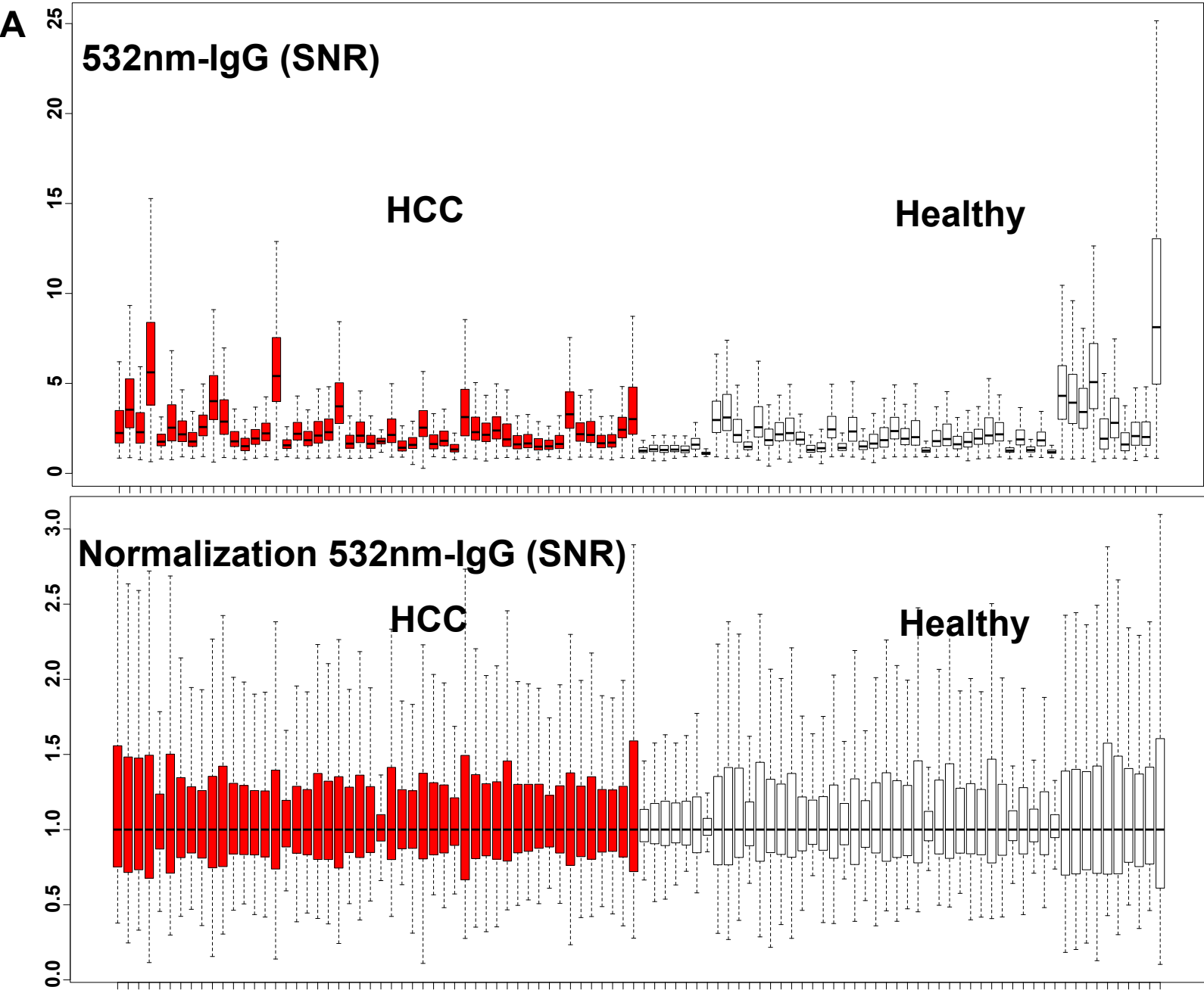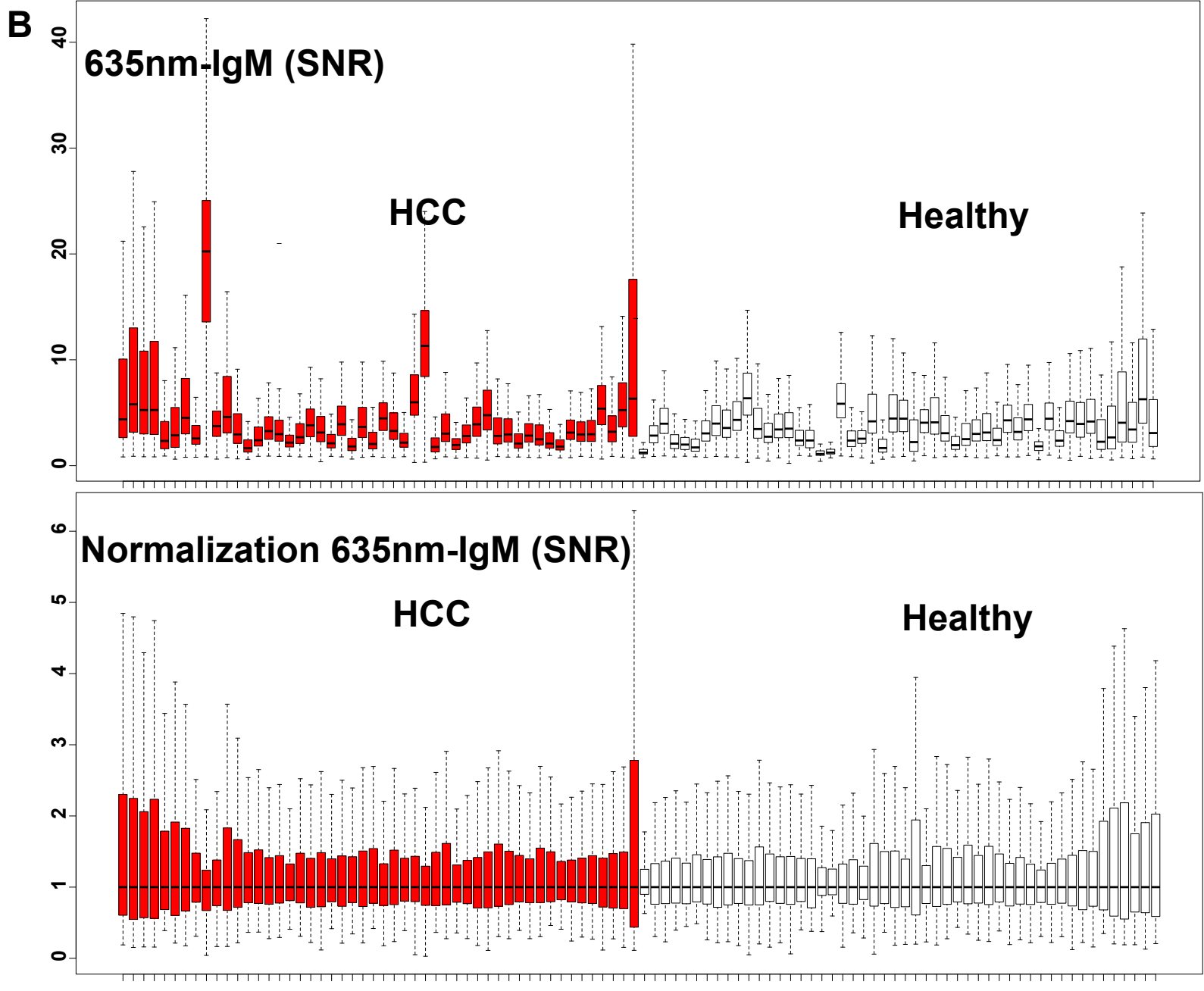

Supplement: Supplementary file 1 — Additional file 1:. Fig. S1. Boxplot of signals obtained from HuProtTM array. In discovery phase (I), boxplot of signal intensities (SNR = median foreground intensity/median background intensity) was shown after normalization. The red boxes represent 50 HCC samples and the other boxes represent 50 healthy controls. The antigens were recognized by human autoantibodies of the IgG (A) and IgM (B) isotypes, respectively. [file 13045_2020_918_MOESM1_ESM.pdf]

Supplementary Fig. S3

A

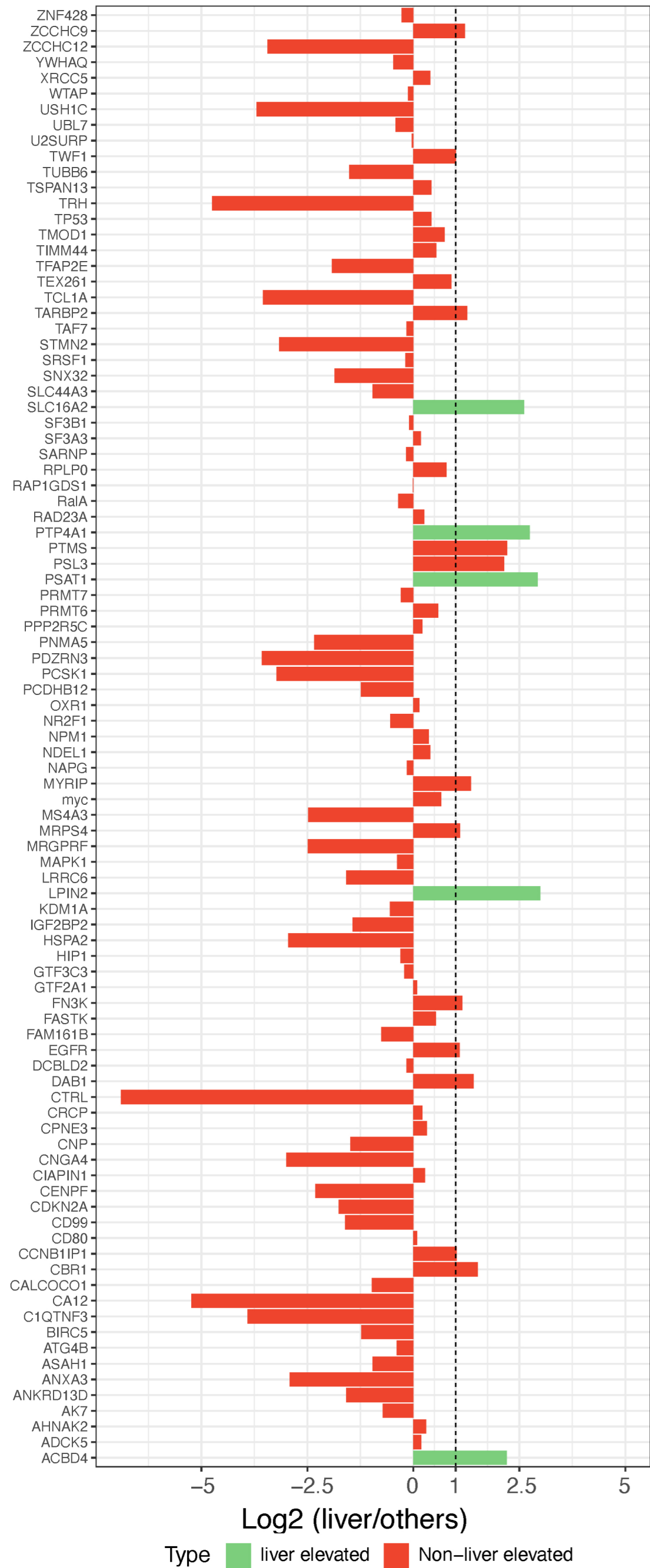

B

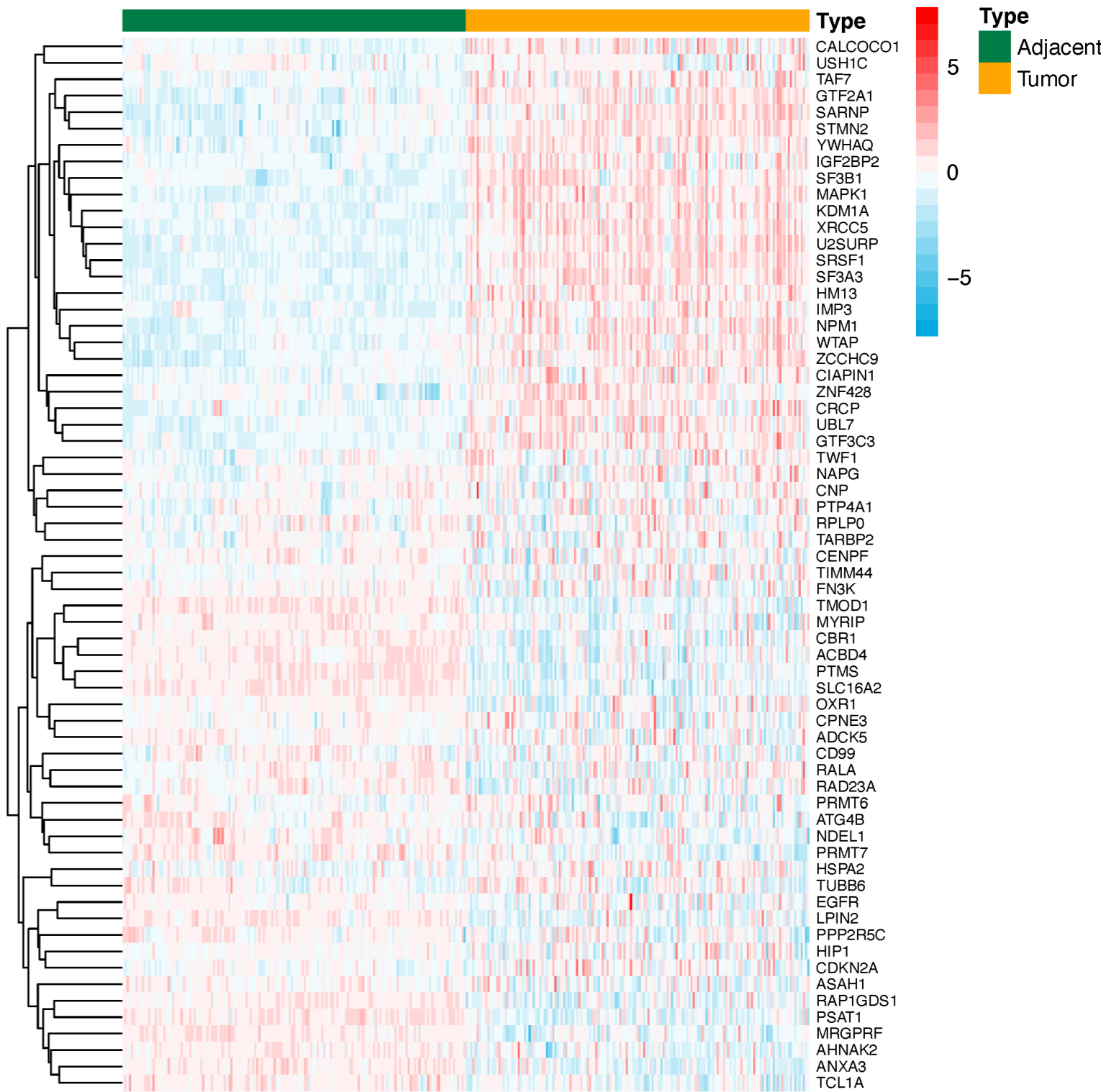

C

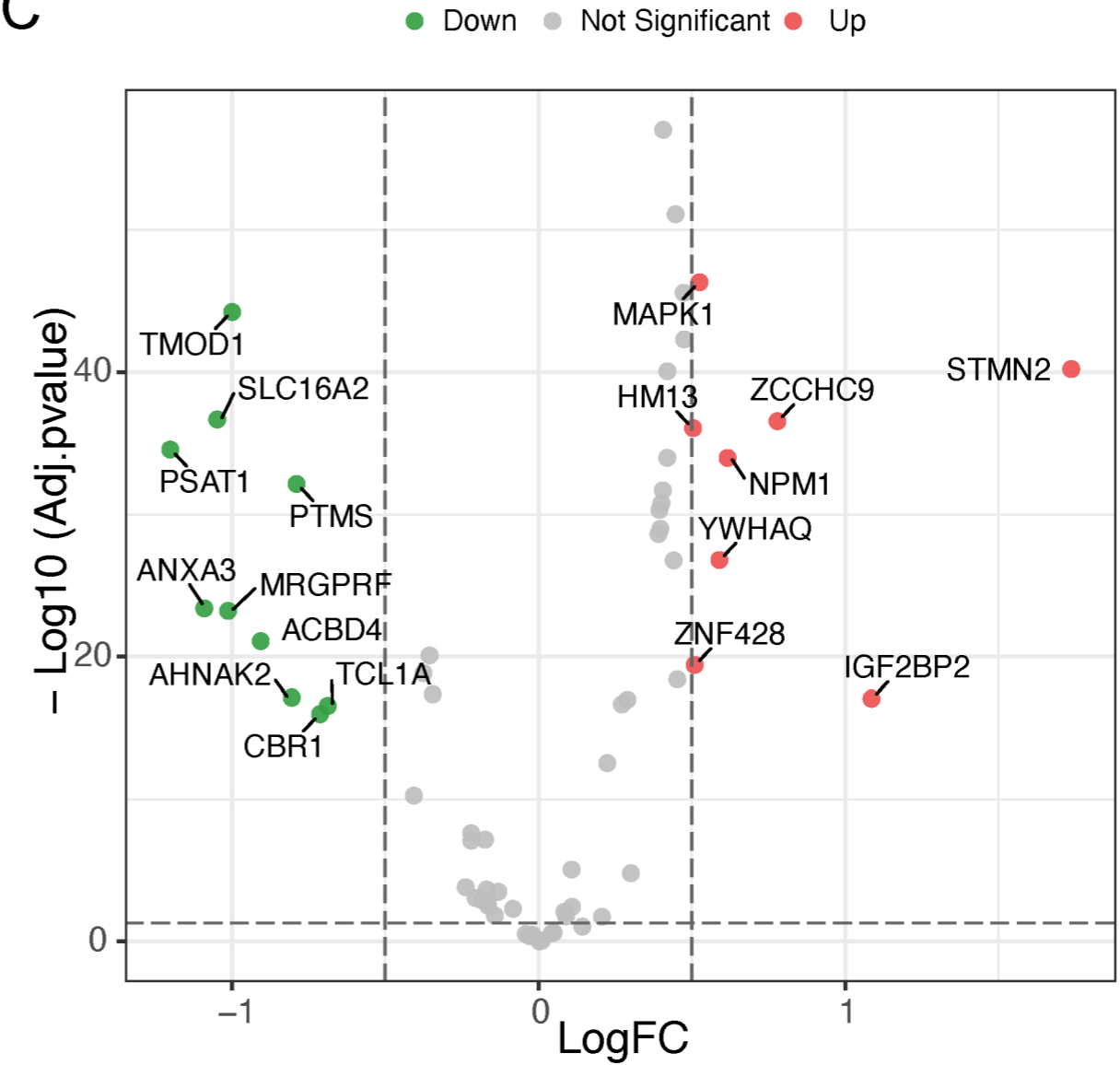

Supplement: Supplementary file 3 — Additional file 3: Fig. S3. Expression level for the 100 proteins. (A) Gene expression level of 100 proteins in the liver compared to other tissues using The Human Protein Atlas (http://www.proteinatlas.org/). Based on this database, 7 proteins were absent in the liver, including MAS1, C3orf56, DCAF4L2, DEFB112, GPR78, PAGE1 and SCGB1C2. 16 genes showed increased mRNA expression level in the liver. Liver elevated proteins reported in the liver-specific proteome of The Human Protein Atlas were labeled with green. (B) Totally, 64 of these 100 proteins were found and further analyzed in paired HCC tumor and adjacent non-tumor liver tissues according to our previous proteomics (Ref. Cell. 179, 561-577 (2019)). (C) Among the 64 proteins, 10 were down-regulated and 8 were up-regulated significantly in HCC tumor, compared with adjacent liver tissues (adjusted p < 0.05 and∣log2 FC∣ > 0.5) according to our previous proteomics (Ref. Cell. 179, 561-577 (2019)). ( 4412 kb) [file 13045_2020_918_MOESM3_ESM.pdf]
