# Supplementary material for: Autoantibody signature in hepatocellular carcinoma using seromics
Source: J Hematol Oncol. 2020 Jul 2;13:85. doi: 10.1186/s13045-020-00918-x (PMC7330948; doi:10.1186/s13045-020-00918-x)

Supplementary Fig. S2

A

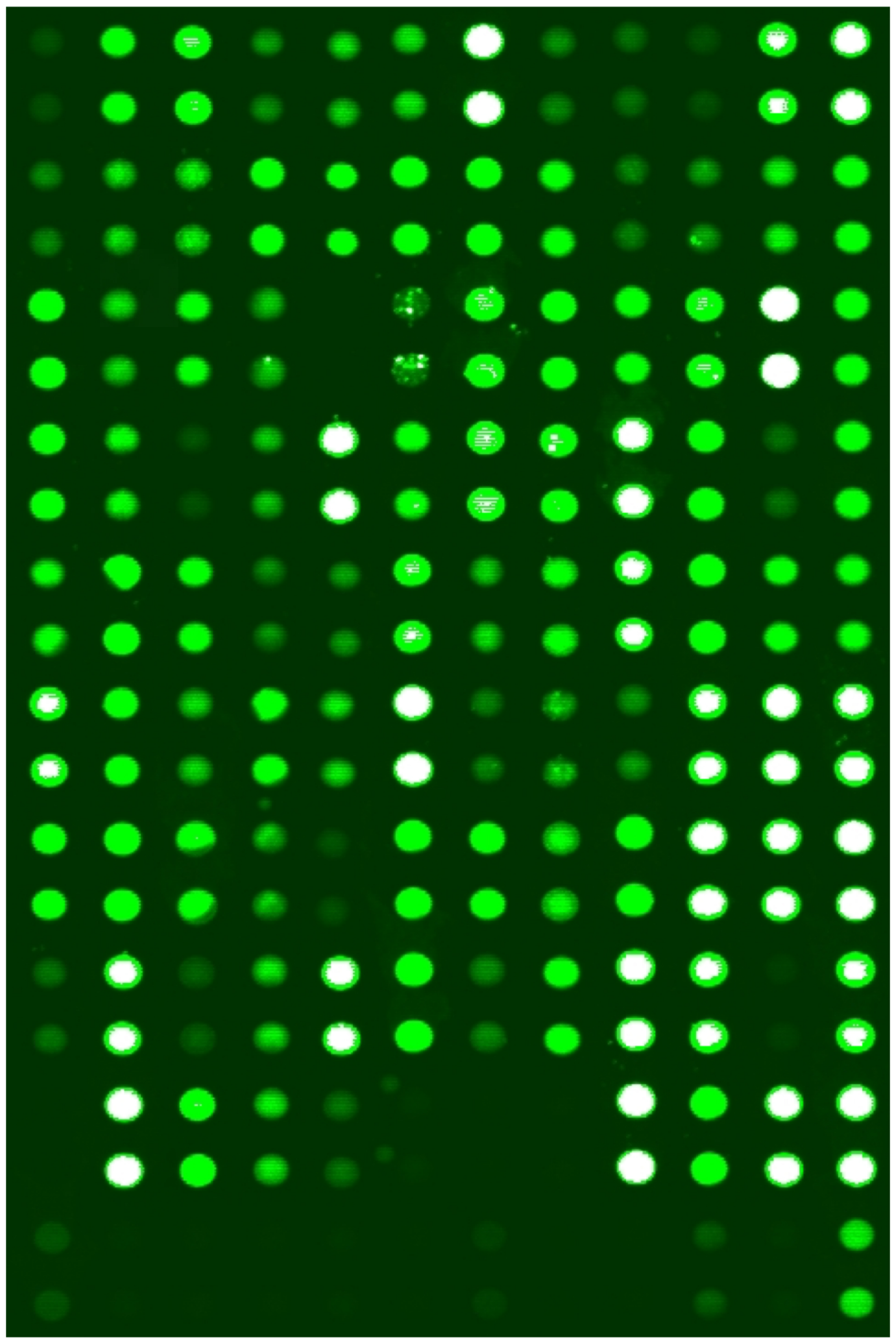

BIRC5	SARNP	TMOD1	TWF1	DEFB112	CBR1	TARBP2	MAS1	ASAH1	PPP2R5C	WTAP	DAB1
PCDHB12	ZCCHC12	CTRL	FN3K	CIAPIN1	ANKRD13D	TCL1A	C3orf56	NR2F1	XRCC5	TAF7	C1QTNF3
PTP4A1	PSL3	GTF2A1	TRH	EGFR	IGF2BP2	TFAP2E	PNMA5	ADCK5_frag	CA12	ZNF428	SLC44A3
USH1C	FAM161B	PCSK1	PDZRN3	AHNAK2	RALA	YWHAQ	LRRC6	CD99	SNX32	U2SURP	NAPG
STMN2	CCNB1IP1	SF3B1	HIP1	SRSF1	CALCOCO1	ZCCHC9	TEX261	PRMT7	TUBB6	TP53	DCAF4L2
RAD23A	CNP	SCGB1C2	KDM1A	NDEL1	GTF3C3	LPIN2	MYRIP	FASTK	AK7	OXR1	RAP1GDS1
NPM1	SF3A3	MS4A3	DCBLD2	MRPS4	ANXA3	CRCP	GPR78	TIMM44	ATG4B	UBL7	PTMS
ACBD4	PSAT1	MRGPRF	CNGA4	HSPA2	TSPAN13	SLC16A2	PRMT6	CPNE3	CD80	myc	MAPK1
Buffer	GST-0.1	GST-0.05	GST-0.02	GST-0.01	Buffer	BSA	Buffer	CDKN2A	RPLP0	CENPF	PAGE1
Landmarker	Buffer	IgM-0.1	IgM-0.05	IgM-0.02	IgM-0.01	Anti-IgM	IgG-0.1	IgG-0.05	IgG-0.02	IgG-0.01	Anti-IgG

B

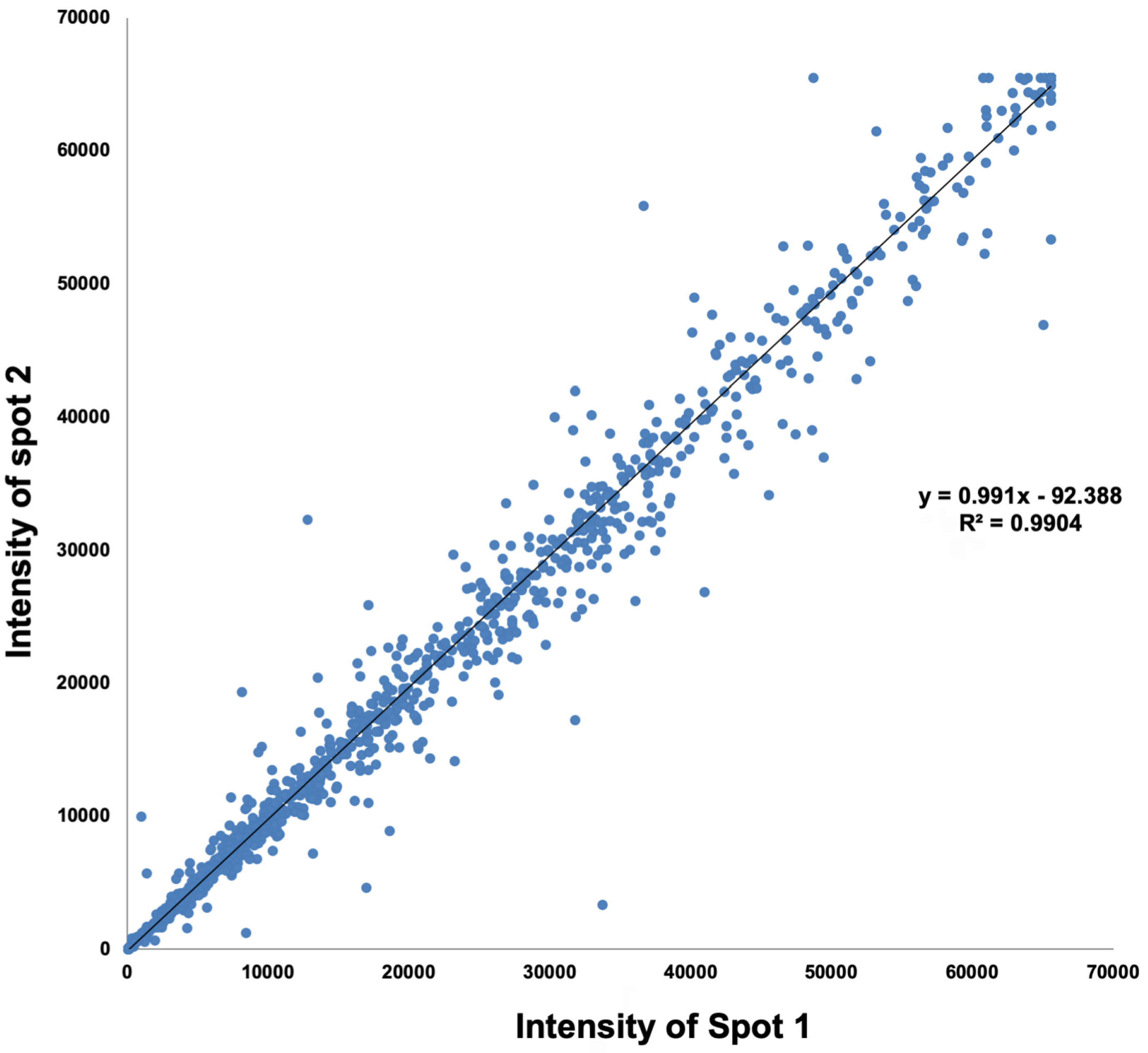

C

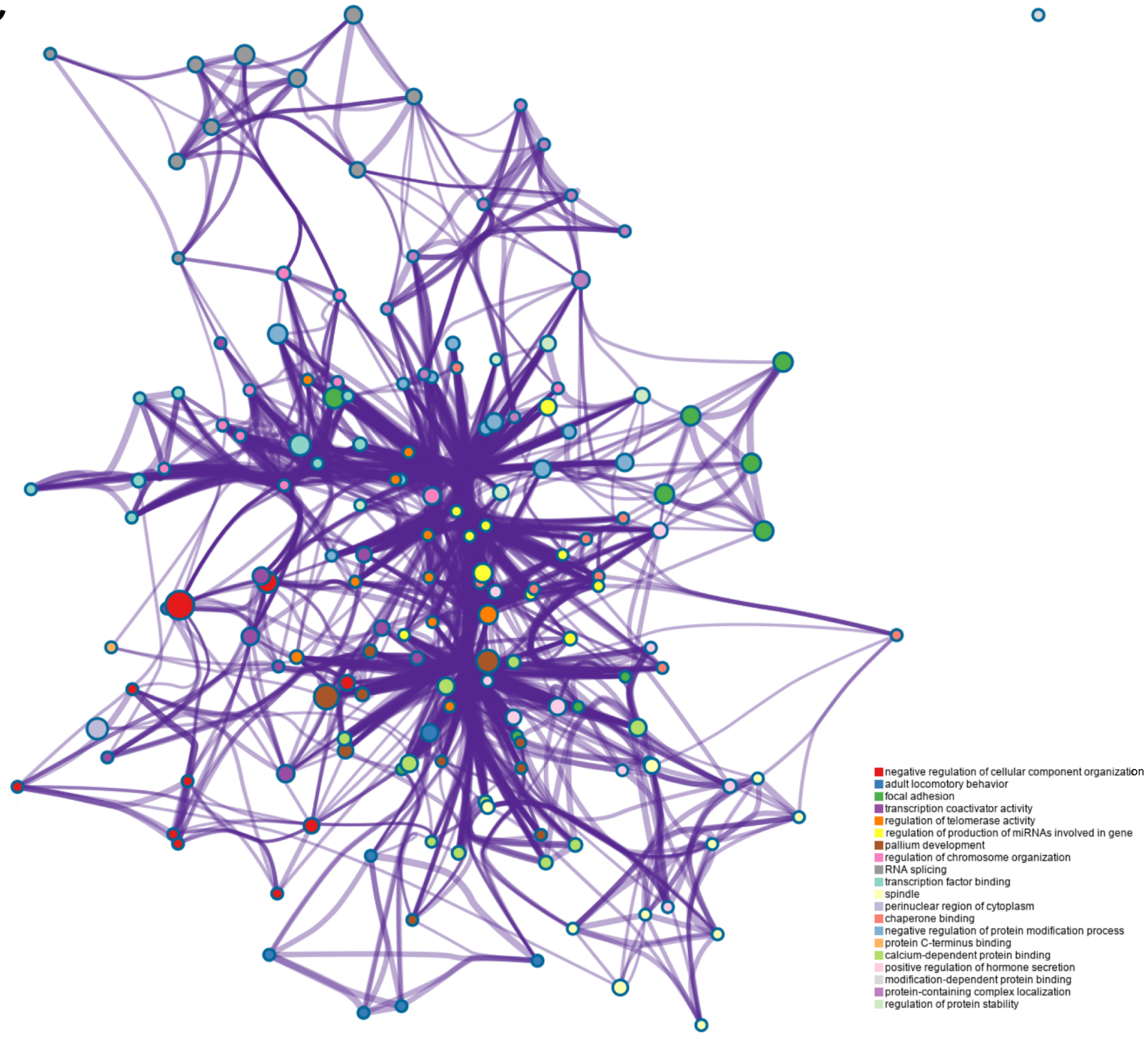

Supplement: Supplementary file 2 — Additional file 2:. Fig. S2. One hundred proteins for HCC Focused Arrays. (A) A total of 100 proteins were selected to prepare HCC Focused Arrays using GST fusion proteins. Anti-GST antibody was employed for quality control of HCC Focused Array. (B) Two spots (spot 1 and spot 2) of each protein were printed onto array with correlation coefficient (R2) approximate of 0.99. (C) Function enrichment analysis identified biological processes, including focal adhesion, and negative regulation of cellular component organization and protein modification process. [file 13045_2020_918_MOESM2_ESM.pdf]
