# Supplementary material for: Autoantibody signature in hepatocellular carcinoma using seromics
Source: J Hematol Oncol. 2020 Jul 2;13:85. doi: 10.1186/s13045-020-00918-x (PMC7330948; doi:10.1186/s13045-020-00918-x)
Supplement: Supplementary file 4 — Additional file 4:. Fig. S4. The representative blots for both HCC and controls. (A) HCC Focused Arrays incubated with HCC, liver cirrhosis (Cirrhotic), and healthy control (Healthy), respectively. (B) Performance of CIAPIN1, EGFR, MAS1, SLC44A3, ASAH1, UBL7 and ZNF428 in the test phase (II). [file 13045_2020_918_MOESM4_ESM.pdf]

A

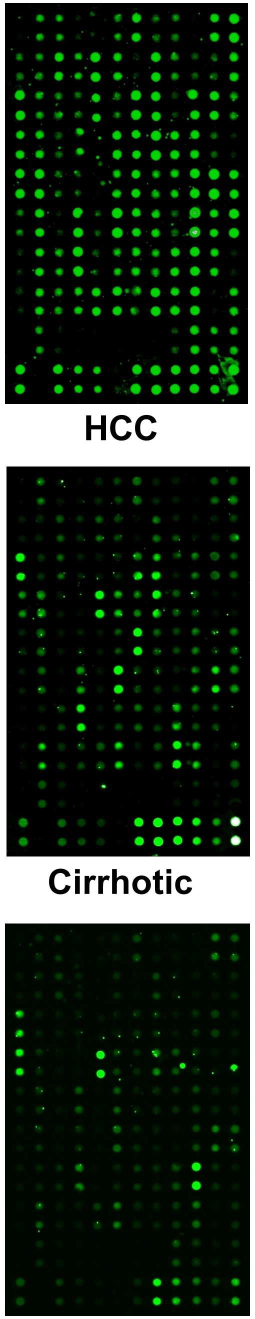

B

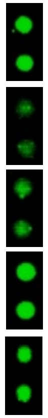
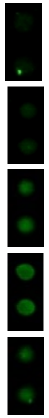

Protein	HCC	Cirrhotic	*p*-value	FC	Sensitivity	Specificity
EGFR			0.00	2.1	26.2%	94.6%
MAS1			0.00	1.3	31.0%	90.2%
SLC44A3			0.00	1.6	24.4%	95.7%
ZNF428			0.00	1.7	18.5%	94.6%
UBL7			0.00	1.9	17.3%	90.2%

C

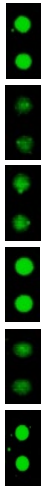
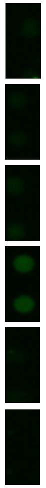

Protein	HCC	Healthy	*p*-value	FC	Sensitivity	Specificity
EGFR			0.00	1.8	25.1%	90.4%
MAS1			0.00	1.4	33.2%	90.4%
SLC44A3			0.00	1.4	21.8%	92.6%
ZNF428			0.00	2.3	23.2%	94.8%
ASAH1			0.00	1.2	19.9%	97.8%
CIAPIN1			0.00	1.7	21.4%	96.3%
