# Supplementary material for: Autoantibody signature in hepatocellular carcinoma using seromics
Source: J Hematol Oncol. 2020 Jul 2;13:85. doi: 10.1186/s13045-020-00918-x (PMC7330948; doi:10.1186/s13045-020-00918-x)
Supplement: Supplementary file 5 — Additional file 5:. Table S1. Summary of the study subjects. [file 13045_2020_918_MOESM5_ESM.docx]

**Table S1. Summary of the study subjects.**

| **Characteristics** | **Discovery phase (I)** | | | **Test phase (II)** | | | **Validation phase (III)** | | |
| --- | --- | --- | --- | --- | --- | --- | --- | --- | --- |
| **Healthy** | **No.** | **Mean±S.D.** | **%** | **No.** | **Mean±S.D.** | **%** | **No.** | **Mean±S.D.** | **%** |
| **Age (years)** |  | 55.37±14.41 |  |  | 46.21±10.80 |  |  | 45.91±10.97 |  |
| **Sex** |  |  |  |  |  |  |  |  |  |
| Male | 31 |  | 62.0 | 102 |  | 62.2 | 117 |  | 65.4 |
| Female | 19 |  | 38.0 | 62 |  | 37.8 | 62 |  | 34.6 |
| **AFP** |  |  |  |  |  |  |  |  |  |
| <20 (ng/mL) | 50 |  | 100.0 | 162 |  | 98.8 | 177 |  | 98.9 |
| ≥20 (ng/mL) | 0 |  | 0.0 | 1 |  | 0.6 | 0 |  | 0.0 |
| Missing | 0 |  | 0.0 | 1 |  | 0.6 | 2 |  | 1.1 |
| **Cirrhotic** | **No.** | **Mean±S.D.** | **%** | **No.** | **Mean±S.D.** | **%** | **No.** | **Mean±S.D.** | **%** |
| **Age (years)** |  |  |  |  | 54.90±13.57 |  |  | 54.62±12.03 |  |
| **Sex** |  |  |  |  |  |  |  |  |  |
| Male |  |  |  | 86 |  | 66.2 | 81 |  | 68.1 |
| Female |  |  |  | 44 |  | 33.8 | 38 |  | 31.9 |
| **AFP** |  |  |  |  |  |  |  |  |  |
| <20 (ng/mL) |  |  |  | 105 |  | 80.8 | 85 |  | 71.4 |
| ≥20 (ng/mL) |  |  |  | 20 |  | 15.4 | 21 |  | 17.7 |
| Missing |  |  |  | 5 |  | 3.8 | 13 |  | 10.9 |
| **HCC** | **No.** | **Mean±S.D.** | **%** | **No.** | **Mean±S.D.** | **%** | **No.** | **Mean±S.D.** | **%** |
| **Age (years)** |  | 58.08±11.19 |  |  | 53.89±12.54 |  |  | 51.57±12.08 |  |
| **Sex** |  |  |  |  |  |  |  |  |  |
| Male | 43 |  | 86.0 | 241 |  | 85.5 | 239 |  | 85.7 |
| Female | 7 |  | 14.0 | 41 |  | 14.5 | 40 |  | 14.3 |
| **AFP** |  |  |  |  |  |  |  |  |  |
| <20 (ng/mL) | 24 |  | 48.0 | 119 |  | 42.2 | 98 |  | 35.1 |
| ≥20 (ng/mL) | 25 |  | 50.0 | 154 |  | 54.6 | 171 |  | 61.3 |
| Missing | 1 |  | 2.0 | 9 |  | 3.2 | 10 |  | 3.6 |
| **Smoking** |  |  |  |  |  |  |  |  |  |
| Yes | 7 |  | 14.0 | 51 |  | 18.1 | 54 |  | 19.4 |
| No | 43 |  | 86.0 | 92 |  | 32.6 | 95 |  | 34.0 |
| Missing | 0 |  | 0.0 | 139 |  | 49.3 | 130 |  | 46.6 |
| **Alcohol** |  |  |  |  |  |  |  |  |  |
| Yes | 2 |  | 4.0 | 44 |  | 15.6 | 47 |  | 16.8 |
| No | 48 |  | 96.0 | 99 |  | 35.1 | 102 |  | 36.6 |
| Missing | 0 |  | 0.0 | 139 |  | 49.3 | 130 |  | 46.6 |
| **ALT** |  |  |  |  |  |  |  |  |  |
| ≤50 (U/L) | 33 |  | 66.0 | 200 |  | 70.9 | 186 |  | 66.7 |
| >50 (U/L) | 8 |  | 16.0 | 58 |  | 20.6 | 61 |  | 21.9 |
| Missing | 9 |  | 18.0 | 24 |  | 8.5 | 32 |  | 11.5 |
| **AST** |  |  |  |  |  |  |  |  |  |
| ≤40 (U/L) | 29 |  | 58.0 | 165 |  | 58.5 | 150 |  | 53.8 |
| >40 (U/L) | 12 |  | 24.0 | 87 |  | 30.9 | 95 |  | 34.1 |
| Missing | 9 |  | 18.0 | 30 |  | 10.6 | 34 |  | 12.2 |
| **HBsAg** |  |  |  |  |  |  |  |  |  |
| Positive | 35 |  | 70.0 | 197 |  | 69.9 | 207 |  | 74.2 |
| Negative | 7 |  | 14.0 | 46 |  | 16.3 | 30 |  | 10.8 |
| Missing | 8 |  | 16.0 | 39 |  | 13.8 | 42 |  | 15.1 |
| **HBeAg** |  |  |  |  |  |  |  |  |  |
| Positive | 7 |  | 14.0 | 43 |  | 15.2 | 51 |  | 18.3 |
| Negative  Missing | 41  2 |  | 82.0  4.0 | 218  21 |  | 77.3  7.5 | 211  17 |  | 75.6  6.1 |
|  |  |  |  |  |  |  |  |  |  |
| **HBV DNA** |  |  |  |  |  |  |  |  |  |
| ≤ 1×10^3^ copy/mL  **>**1×10^3^ copy/mL  Missing | 11 22.0  8 16.0  31 62.0 | | | 136 | 48.2 | | 123 | 44.1 | |
|  |  |  |  | 93  53 | 33.0  18.8 | | 100 35.8  56 20.1 | | |
| **Tumor Size** |  |  |  |  |  |  |  |  |  |
| ≤2.0cm | 10 |  | 20.0 | 40 |  | 14.2 | 37 |  | 13.3 |
| >2.0cm | 40 |  | 80.0 | 211 |  | 74.8 | 193 |  | 69.2 |
| Missing | 0 |  | 0.0 | 31 |  | 11.0 | 49 |  | 17.6 |
| **BCLC Stage** |  |  |  |  |  |  |  |  |  |
| 0 | 10 |  | 20.0 | 22 |  | 7.8 | 27 |  | 9.7 |
| A | 5 |  | 10.0 | 38 |  | 13.5 | 32 |  | 11.5 |
| B | 19 |  | 38.0 | 152 |  | 53.9 | 121 |  | 43.4 |
| C | 4 |  | 8.0 | 37 |  | 13.1 | 51 |  | 18.3 |
| D | 0 |  | 0.0 | 1 |  | 0.4 | 1 |  | 0.4 |
| Missing | 12 |  | 24.0 | 32 |  | 11.3 | 47 |  | 16.8 |
| **Tumor Number** |  |  |  |  |  |  |  |  |  |
| 1 | 42 |  | 84.0 | 183 |  | 64.9 | 171 |  | 61.3 |
| 2 | 4 |  | 8.0 | 33 |  | 11.7 | 26 |  | 9.3 |
| 3 | 1 |  | 2.0 | 6 |  | 2.1 | 6 |  | 2.2 |
| 4 | 0 |  | 0.0 | 1 |  | 0.4 | 1 |  | 0.4 |
| 8 | 0 |  | 0.0 | 0 |  | 0.0 | 1 |  | 0.4 |
| Mutiple | 3 |  | 6.0 | 28 |  | 9.9 | 29 |  | 10.4 |
| Missing | 0 |  | 0.0 | 31 |  | 11.0 | 45 |  | 16.1 |
| **Edmondson Grade** |  |  |  |  |  |  |  |  |  |
| Ⅰ | 1 |  | 2.0 | 3 |  | 1.1 | 4 |  | 1.4 |
| Ⅱ | 19 |  | 38.0 | 67 |  | 23.8 | 62 |  | 22.2 |
| Ⅲ | 7 |  | 14.0 | 105 |  | 37.2 | 83 |  | 29.7 |
| Ⅰ-Ⅱ | 1 |  | 2.0 | 3 |  | 1.1 | 2 |  | 0.7 |
| Ⅱ-Ⅲ | 15 |  | 30.0 | 33 |  | 11.7 | 40 |  | 14.3 |
| Missing | 7 |  | 14.0 | 71 |  | 25.2 | 88 |  | 31.5 |
| **Child Pugh Grading** |  |  |  |  |  |  |  |  |  |
| A | 36 |  | 72.0 | 231 |  | 81.9 | 220 |  | 78.9 |
| B | 2 |  | 4.0 | 21 |  | 7.4 | 16 |  | 5.7 |
| C | 0 |  | 0.0 | 1 |  | 0.4 | 2 |  | 0.7 |
| Missing | 12 |  | 24.0 | 29 |  | 10.3 | 41 |  | 14.7 |
| **TNM Stage** |  |  |  |  |  |  |  |  |  |
| IA | 10 |  | 20.0 | 25 |  | 8.9 | 26 |  | 9.3 |
| IB | 29 |  | 58.0 | 136 |  | 48.2 | 109 |  | 39.1 |
| II | 2 |  | 4.0 | 36 |  | 12.8 | 37 |  | 13.3 |
| ⅢA | 2 |  | 4.0 | 18 |  | 6.4 | 10 |  | 3.6 |
| ⅢB | 2 |  | 4.0 | 17 |  | 6.0 | 23 |  | 8.2 |
| ⅣA | 4 |  | 8.0 | 19 |  | 6.7 | 18 |  | 6.5 |
| ⅣB | 1 |  | 2.0 | 1 |  | 0.4 | 9 |  | 3.2 |
| Missing | 0 |  | 0.0 | 30 |  | 10.6 | 47 |  | 16.8 |
| **Chinese HCC Stage** |  |  |  |  |  |  |  |  |  |
| Ⅰa | 19 |  | 38.0 | 99 |  | 35.1 | 82 |  | 29.4 |
| Ⅰb | 13 |  | 26.0 | 70 |  | 24.8 | 61 |  | 21.9 |
| Ⅱa | 2 |  | 4.0 | 23 |  | 8.2 | 20 |  | 7.2 |
| Ⅱb | 0 |  | 0.0 | 20 |  | 7.1 | 17 |  | 6.1 |
| Ⅲa | 2 |  | 4.0 | 17 |  | 6.0 | 23 |  | 8.2 |
| Ⅲb | 2 |  | 4.0 | 20 |  | 7.1 | 25 |  | 9.0 |
| Ⅳ | 0 |  | 0.0 | 1 |  | 0.4 | 2 |  | 0.7 |
| Missing | 12 |  | 24.0 | 32 |  | 11.3 | 49 |  | 17.6 |
