# Supplementary material for: Autoantibody signature in hepatocellular carcinoma using seromics
Source: J Hematol Oncol. 2020 Jul 2;13:85. doi: 10.1186/s13045-020-00918-x (PMC7330948; doi:10.1186/s13045-020-00918-x)
Supplement: Supplementary file 6 — Additional file 6:. Table S2. Performance of 55 potential biomarkers in the test phase (II). [file 13045_2020_918_MOESM6_ESM.docx]

**Table S2. Performance of 55 potential biomarkers in the test phase (II).**

| **Protein** | **HCC *vs.* Healthy** | | | | **HCC *vs.* Cirrhotic** | | | |
| --- | --- | --- | --- | --- | --- | --- | --- | --- |
|  | ***p*-value** | **FC** | **Sensitivity** | **Specificity** | ***p*-value** | **FC** | **Sensitivity** | **Specificity** |
| ASAH1 | 0.00 | 1.2 | 19.9% | 97.8% |  |  | / |  |
| CIAPIN1 | 0.00 | 1.7 | 21.4% | 96.3% |  |  | / |  |
| ANKRD13D | 0.00 | 1.9 | 30.6% | 97.8% |  |  | / |  |
| CA12 | 0.02 | 1.4 | 18.5% | 94.1% |  |  | / |  |
| CCNB1IP1 | 0.01 | 1.5 | 15.5% | 91.1% |  |  | / |  |
| CD80 | 0.00 | 1.5 | 24.0% | 91.1% |  |  | / |  |
| CNP | 0.00 | 1.3 | 17.0% | 99.3% |  |  | / |  |
| CPNE3 | 0.02 | 1.4 | 18.5% | 91.1% |  |  | / |  |
| DAB1 | 0.01 | 1.5 | 19.6% | 91.1% |  |  | / |  |
| DCAF4L2 | 0.00 | 2.2 | 23.6% | 95.6% |  |  | / |  |
| GTF3C3 | 0.00 | 1.5 | 22.5% | 90.4% |  |  | / |  |
| KDM1A | 0.00 | 2.2 | 22.9% | 95.6% |  |  | / |  |
| MAPK1 | 0.00 | 1.5 | 25.5% | 91.9% |  |  | / |  |
| MS4A3 | 0.00 | 1.3 | 18.5% | 97.8% |  |  | / |  |
| NDEL1 | 0.00 | 1.5 | 25.1% | 91.9% |  |  | / |  |
| NPM1 | 0.01 | 3.3 | 19.9% | 96.3% |  |  | / |  |
| PCDHB12 | 0.00 | 1.2 | 18.1% | 95.6% |  |  | / |  |
| PNMA5 | 0.01 | 1.7 | 24.4% | 90.4% |  |  | / |  |
| PRMT7 | 0.03 | 1.7 | 17.0% | 93.3% |  |  | / |  |
| PSAT1 | 0.00 | 1.4 | 21.4% | 90.4% |  |  | / |  |
| RPLP0 | 0.01 | 1.3 | 20.7% | 90.4% |  |  | / |  |
| TMOD1 | 0.00 | 2.5 | 32.8% | 92.6% |  |  | / |  |
| TUBB6 | 0.00 | 1.3 | 15.5% | 98.5% |  |  | / |  |
| TWF1 | 0.00 | 1.2 | 21.0% | 91.1% |  |  | / |  |
| UBL7 |  |  | / |  | 0.00 | 1.9 | 17.3% | 90.2% |
| ADCK5 |  |  | / |  | 0.00 | 1.2 | 19.9% | 94.6% |
| ATG4B |  |  | / |  | 0.00 | 1.5 | 19.9% | 91.3% |
| C3orf56 |  |  | / |  | 0.00 | 1.4 | 17.3% | 97.8% |
| CD99 |  |  | / |  | 0.00 | 1.4 | 37.3% | 90.2% |
| CTRL |  |  | / |  | 0.00 | 1.3 | 19.9% | 90.2% |
| FAM161B |  |  | / |  | 0.00 | 1.5 | 18.5% | 93.5% |
| HIP1 |  |  | / |  | 0.00 | 1.4 | 19.6% | 95.7% |
| LPIN2 |  |  | / |  | 0.00 | 1.4 | 29.2% | 90.2% |
| NR2F1 |  |  | / |  | 0.00 | 1.3 | 23.2% | 91.3% |
| PTP4A1 |  |  | / |  | 0.01 | 1.2 | 21.4% | 92.4% |
| RAP1GDS1 |  |  | / |  | 0.00 | 1.5 | 29.9% | 91.3% |
| SF3B1 |  |  | / |  | 0.00 | 1.4 | 16.2% | 91.3% |
| SLC16A2 |  |  | / |  | 0.00 | 1.3 | 24.4% | 90.2% |
| SNX32 |  |  | / |  | 0.00 | 1.3 | 22.1% | 92.4% |
| TIMM44 |  |  | / |  | 0.00 | 1.3 | 15.9% | 97.8% |
| WTAP |  |  | / |  | 0.00 | 2.2 | 28.8% | 90.2% |
| EGFR | 0.00 | 1.8 | 25.1% | 90.4% | 0.00 | 2.1 | 26.2% | 94.6% |
| MAS1 | 0.00 | 1.4 | 33.2% | 90.4% | 0.00 | 1.3 | 31.0% | 90.2% |
| SLC44A3 | 0.00 | 1.4 | 21.8% | 92.6% | 0.00 | 1.6 | 24.4% | 95.7% |
| ZNF428 | 0.00 | 2.3 | 23.2% | 94.8% | 0.00 | 1.7 | 18.5% | 94.6% |
| C1QTNF3 | 0.00 | 1.4 | 29.5% | 91.1% | 0.00 | 1.4 | 26.6% | 90.2% |
| CALCOCO1 | 0.02 | 1.3 | 15.1% | 90.4% | 0.00 | 1.5 | 27.7% | 92.4% |
| DCBLD2 | 0.00 | 1.3 | 16.2% | 93.3% | 0.01 | 1.3 | 16.2% | 95.7% |
| GPR78 | 0.00 | 1.3 | 16.6% | 97.0% | 0.00 | 1.3 | 22.5% | 93.5% |
| NAPG | 0.04 | 1.3 | 19.6% | 91.1% | 0.01 | 1.4 | 22.1% | 93.5% |
| PTMS | 0.00 | 2.2 | 15.5% | 94.1% | 0.01 | 2.0 | 15.5% | 94.6% |
| SARNP | 0.00 | 1.3 | 19.6% | 96.3% | 0.00 | 1.4 | 15.1% | 98.9% |
| SRSF1 | 0.00 | 1.2 | 18.5% | 97.0% | 0.00 | 1.3 | 21.0% | 90.2% |
| TRH | 0.00 | 1.3 | 21.8% | 94.8% | 0.00 | 1.2 | 19.2% | 94.6% |
| TSPAN13 | 0.00 | 1.3 | 18.1% | 96.3% | 0.00 | 1.5 | 23.2% | 95.7% |
