# Supplementary material for: Autoantibody signature in hepatocellular carcinoma using seromics
Source: J Hematol Oncol. 2020 Jul 2;13:85. doi: 10.1186/s13045-020-00918-x (PMC7330948; doi:10.1186/s13045-020-00918-x)
Supplement: Supplementary file 7 — Additional file 7:. Table S3. Performance of the 7-AAb panel and AFP in HBsAg+/HBsAg--HCC detection. [file 13045_2020_918_MOESM7_ESM.docx]

**Table S3. Performance of the 7-AAb panel and AFP in HBsAg^+^/ HBsAg^-^-HCC detection.**

| **HBsAg** | **Detection^a^** | **Test Phase (II)** | | | **Validation Phase (III)** | | | **Test Phase (II)+ Validation Phase (III)** | | |
| --- | --- | --- | --- | --- | --- | --- | --- | --- | --- | --- |
|  |  | **AFP** | **ANN** | **AFP+ANN** | **AFP** | **ANN** | **AFP+ANN** | **AFP** | **ANN** | **AFP+ANN** |
| HBsAg**^+^**-HCC vs. Healthy+ Cirrhotic | AUC | 0.832 | 0.897 | 0.932 | 0.867 | 0.907 | 0.934 | 0.850 | 0.902 | 0.932 |
|  | Specificity | 98.7% | 92.1% | 90.7% | 99.6% | 90.1% | 90.1% | 99.1% | 90.2% | 93.7% |
|  | Sensitivity | 31.4% | 68.1% | 80.9% | 34.7% | 74.0% | 82.1% | 32.8% | 70.6% | 77.3% |
| HBsAg**^+^**-HCC vs. Healthy | AUC | 0.849 | 0.936 | 0.965 | 0.870 | 0.933 | 0.954 | 0.860 | 0.934 | 0.959 |
|  | Specificity | 100.0% | 93.3% | 93.3% | 100.0% | 93.4% | 93.4% | 100.0% | 95.5% | 95.5% |
|  | Sensitivity | 31.4% | 78.2% | 88.8% | 34.7% | 79.1% | 84.7% | 32.8% | 71.6% | 82.6% |
| HBsAg**^+^**-HCC vs. Cirrhotic | AUC | 0.807 | 0.840 | 0.883 | 0.860 | 0.860 | 0.896 | 0.833 | 0.850 | 0.888 |
|  | Specificity | 96.7% | 90.2% | 92.4% | 98.8% | 85.2% | 93.8% | 97.7% | 89.0% | 91.3% |
|  | Sensitivity | 31.4% | 59.6% | 71.8% | 34.7% | 70.9% | 74.5% | 32.8% | 65.6% | 73.2% |
| HBsAg**^-^**-HCC vs. Healthy+ Cirrhotic | AUC | 0.640 | 0.897 | 0.895 | 0.583 | 0.875 | 0.920 | 0.618 | 0.889 | 0.906 |
|  | Specificity | 99.1% | 94.7% | 93.4% | 99.6% | 90.1% | 90.1% | 99.3% | 96.3% | 95.4% |
|  | Sensitivity | 13.3% | 73.3% | 75.6% | 16.7% | 73.3% | 80.0% | 14.7% | 69.3% | 73.3% |
| HBsAg**^-^**-HCC vs. Healthy | AUC | 0.635 | 0.932 | 0.938 | 0.567 | 0.903 | 0.945 | 0.607 | 0.921 | 0.940 |
|  | Specificity | 100.0% | 94.8% | 94.8% | 100.0% | 94.7% | 94.7% | 100.0% | 94.4% | 94.4% |
|  | Sensitivity | 13.3% | 77.8% | 80.0% | 16.7% | 73.3% | 80.0% | 14.7% | 76.0% | 80.0% |
| HBsAg**^-^**-HCC vs. Cirrhotic | AUC | 0.647 | 0.845 | 0.832 | 0.613 | 0.822 | 0.872 | 0.636 | 0.837 | 0.850 |
|  | Specificity | 97.8% | 95.7% | 92.4% | 98.8% | 96.3% | 95.1% | 98.3% | 97.1% | 94.8% |
|  | Sensitivity | 13.3% | 68.9% | 71.1% | 16.7% | 63.3% | 70.0% | 14.7% | 65.3% | 69.3% |

**^a^**The diagnostic cutoff value of AFP was 400 ng/mL.
