# Supplementary material for: Autoantibody signature in hepatocellular carcinoma using seromics
Source: J Hematol Oncol. 2020 Jul 2;13:85. doi: 10.1186/s13045-020-00918-x (PMC7330948; doi:10.1186/s13045-020-00918-x)
Supplement: Supplementary file 8 — Additional file 8:. Table S4. Performance of the 7-AAb panel and AFP to detect HCC with different TNM stages. [file 13045_2020_918_MOESM8_ESM.docx]

**Table S4. Performance of the 7-AAb panel and AFP to detect HCC with different TNM stages.**

| **TNM stage** | **Detection^a^** | **Test Phase (II)** | | | **Validation Phase (III)** | | | **Test Phase (II)+ Validation Phase (III)** | | |
| --- | --- | --- | --- | --- | --- | --- | --- | --- | --- | --- |
|  |  | **AFP** | **ANN** | **AFP+ANN** | **AFP** | **ANN** | **AFP+ANN** | **AFP** | **ANN** | **AFP+ANN** |
| TNM(I) vs. Healthy+ Cirrhotic | AUC | 0.784 | 0.903 | 0.921 | 0.807 | 0.919 | 0.938 | 0.795 | 0.911 | 0.929 |
|  | Specificity | 98.7% | 92.1% | 92.1% | 99.6% | 89.2% | 90.1% | 99.1% | 89.1% | 89.3% |
|  | Sensitivity | 25.0% | 71.7% | 78.3% | 26.6% | 77.3% | 83.6% | 25.7% | 76.1% | 82.1% |
| TNM(I) vs. Healthy | AUC | 0.795 | 0.939 | 0.957 | 0.808 | 0.942 | 0.957 | 0.800 | 0.940 | 0.956 |
|  | Specificity | 100.0% | 96.3% | 96.3% | 100.0% | 93.4% | 93.4% | 100.0% | 93.4% | 93.4% |
|  | Sensitivity | 25.0% | 76.3% | 84.2% | 26.6% | 81.3% | 86.7% | 25.7% | 79.3% | 86.1% |
| TNM(I) vs. Cirrhotic | AUC | 0.768 | 0.851 | 0.868 | 0.805 | 0.877 | 0.901 | 0.785 | 0.863 | 0.883 |
|  | Specificity | 96.7% | 90.2% | 91.3% | 98.8% | 97.5% | 96.3% | 97.7% | 90.8% | 90.8% |
|  | Sensitivity | 25.0% | 66.4% | 71.7% | 26.6% | 65.6% | 74.2% | 25.7% | 67.1% | 75.0% |
| TNM(II) vs. Healthy+ Cirrhotic | AUC | 0.795 | 0.904 | 0.901 | 0.848 | 0.858 | 0.887 | 0.822 | 0.881 | 0.894 |
|  | Specificity | 98.7% | 89.9% | 91.2% | 99.6% | 90.1% | 90.1% | 99.1% | 90.0% | 89.3% |
|  | Sensitivity | 8.3% | 72.2% | 72.2% | 19.4% | 66.7% | 72.2% | 13.9% | 69.4% | 75.0% |
| TNM(II) vs. Healthy | AUC | 0.814 | 0.942 | 0.945 | 0.847 | 0.891 | 0.918 | 0.830 | 0.917 | 0.931 |
|  | Specificity | 100.0% | 93.3% | 94.8% | 100.0% | 90.1% | 90.1% | 100.0% | 93.4% | 93.7% |
|  | Sensitivity | 8.3% | 83.3% | 83.3% | 19.4% | 77.8% | 80.6% | 13.9% | 77.8% | 79.2% |
| TNM(II) vs. Cirrhotic | AUC | 0.766 | 0.849 | 0.835 | 0.852 | 0.797 | 0.829 | 0.809 | 0.823 | 0.833 |
|  | Specificity | 96.7% | 90.2% | 94.6% | 98.8% | 91.4% | 91.4% | 97.7% | 89.6% | 89.6% |
|  | Sensitivity | 8.3% | 58.3% | 58.3% | 19.4% | 58.3% | 66.7% | 13.9% | 53.8% | 61.1% |
| TNM(III) vs. Healthy+ Cirrhotic | AUC | 0.896 | 0.878 | 0.948 | 0.935 | 0.895 | 0.948 | 0.915 | 0.888 | 0.949 |
|  | Specificity | 98.7% | 89.9% | 92.5% | 99.6% | 95.3% | 95.3% | 99.1% | 93.0% | 93.7% |
|  | Sensitivity | 48.5% | 69.7% | 87.9% | 56.3% | 71.9% | 84.4% | 52.3% | 69.2% | 86.2% |
| TNM(III) vs. Healthy | AUC | 0.915 | 0.921 | 0.974 | 0.943 | 0.923 | 0.965 | 0.928 | 0.922 | 0.970 |
|  | Specificity | 100.0% | 93.3% | 96.3% | 100.0% | 93.4% | 97.4% | 100.0% | 93.4% | 97.6% |
|  | Sensitivity | 48.5% | 78.8% | 90.9% | 56.3% | 75.0% | 84.4% | 52.3% | 76.9% | 86.2% |
| TNM(III) vs. Cirrhotic | AUC | 0.868 | 0.815 | 0.911 | 0.921 | 0.842 | 0.916 | 0.895 | 0.831 | 0.914 |
|  | Specificity | 96.7% | 84.8% | 95.7% | 98.8% | 91.4% | 98.8% | 97.7% | 90.2% | 97.1% |
|  | Sensitivity | 48.5% | 66.7% | 78.8% | 56.3% | 71.9% | 81.3% | 52.3% | 66.2% | 80.0% |
| TNM(IV) vs. Healthy+ Cirrhotic | AUC | 0.837 | 0.896 | 0.944 | 0.855 | 0.891 | 0.965 | 0.846 | 0.893 | 0.955 |
|  | Specificity | 98.7% | 96.5% | 96.5% | 99.6% | 96.6% | 96.6% | 99.1% | 95.0% | 94.3% |
|  | Sensitivity | 40.0% | 60.0% | 80.0% | 44.0% | 72.0% | 84.0% | 42.2% | 66.7% | 82.2% |
| TNM(IV) vs. Healthy | AUC | 0.854 | 0.939 | 0.974 | 0.859 | 0.915 | 0.980 | 0.856 | 0.926 | 0.977 |
|  | Specificity | 100.0% | 92.6% | 93.3% | 100.0% | 94.7% | 94.7% | 100.0% | 93.0% | 93.4% |
|  | Sensitivity | 40.0% | 75.0% | 90.0% | 44.0% | 76.0% | 88.0% | 42.2% | 75.6% | 88.9% |
| TNM(IV) vs. Cirrhotic | AUC | 0.813 | 0.833 | 0.899 | 0.847 | 0.844 | 0.938 | 0.831 | 0.839 | 0.917 |
|  | Specificity | 96.7% | 93.8% | 93.8% | 98.8% | 92.4% | 92.4% | 97.7% | 90.2% | 89.6% |
|  | Sensitivity | 40.0% | 72.0% | 84.0% | 44.0% | 60.0% | 80.0% | 42.2% | 66.7% | 80.0% |

**^a^**The diagnostic cutoff value of AFP was 400 ng/mL.
