# Supplementary material for: Autoantibody signature in hepatocellular carcinoma using seromics
Source: J Hematol Oncol. 2020 Jul 2;13:85. doi: 10.1186/s13045-020-00918-x (PMC7330948; doi:10.1186/s13045-020-00918-x)
Supplement: Supplementary file 9 — Additional file 9:. Table S5. Performance of the 7-AAb panel and AFP to detect HCC with different Chinese HCC stages. [file 13045_2020_918_MOESM9_ESM.docx]

**Table S5. Performance of the 7-AAb panel and AFP to detect HCC with different Chinese HCC stages.**

| **Chinese stage** | **Detection^a^** | **Test Phase (II)** | | | **Validation Phase (III)** | | | **Test Phase (II)+ Validation Phase (III)** | | |
| --- | --- | --- | --- | --- | --- | --- | --- | --- | --- | --- |
|  |  | **AFP** | **ANN** | **AFP+ANN** | **AFP** | **ANN** | **AFP+ANN** | **AFP** | **ANN** | **AFP+ANN** |
| Chinese(I) vs. Healthy+ Cirrhotic | AUC | 0.790 | 0.906 | 0.923 | 0.810 | 0.911 | 0.928 | 0.799 | 0.909 | 0.926 |
|  | Specificity | 98.7% | 89.9% | 90.7% | 99.6% | 90.1% | 90.1% | 99.1% | 89.1% | 89.3% |
|  | Sensitivity | 24.4% | 73.1% | 78.8% | 24.3% | 75.0% | 82.4% | 24.3% | 75.7% | 81.8% |
| Chinese(I) vs. Healthy | AUC | 0.801 | 0.942 | 0.959 | 0.811 | 0.935 | 0.949 | 0.806 | 0.938 | 0.954 |
|  | Specificity | 100.0% | 96.3% | 96.3% | 100.0% | 93.4% | 93.4% | 100.0% | 93.4% | 93.4% |
|  | Sensitivity | 24.4% | 76.3% | 84.4% | 24.3% | 80.1% | 85.3% | 24.3% | 79.1% | 85.5% |
| Chinese(I) vs. Cirrhotic | AUC | 0.772 | 0.853 | 0.870 | 0.809 | 0.866 | 0.889 | 0.789 | 0.860 | 0.879 |
|  | Specificity | 96.7% | 90.2% | 92.4% | 98.8% | 91.4% | 91.4% | 97.7% | 91.9% | 91.3% |
|  | Sensitivity | 24.4% | 65.6% | 70.6% | 24.3% | 66.9% | 74.3% | 24.3% | 65.2% | 72.6% |
| Chinese(II) vs. Healthy+ Cirrhotic | AUC | 0.834 | 0.860 | 0.902 | 0.834 | 0.861 | 0.908 | 0.835 | 0.861 | 0.905 |
|  | Specificity | 98.7% | 89.9% | 91.2% | 99.6% | 90.1% | 90.1% | 99.1% | 90.2% | 89.5% |
|  | Sensitivity | 25.6% | 65.1% | 76.7% | 30.6% | 63.9% | 72.2% | 27.8% | 63.3% | 75.9% |
| Chinese(II) vs. Healthy | AUC | 0.851 | 0.907 | 0.943 | 0.831 | 0.896 | 0.938 | 0.841 | 0.901 | 0.939 |
|  | Specificity | 100.0% | 93.3% | 93.3% | 100.0% | 90.1% | 90.1% | 100.0% | 93.4% | 93.4% |
|  | Sensitivity | 25.6% | 76.7% | 86.0% | 30.6% | 75.0% | 80.6% | 27.8% | 73.4% | 81.0% |
| Chinese(II) vs. Cirrhotic | AUC | 0.808 | 0.790 | 0.842 | 0.841 | 0.797 | 0.854 | 0.824 | 0.794 | 0.849 |
|  | Specificity | 96.7% | 84.8% | 83.7% | 98.8% | 85.2% | 85.2% | 97.7% | 86.1% | 85.0% |
|  | Sensitivity | 25.6% | 60.5% | 74.4% | 30.6% | 61.1% | 69.4% | 27.8% | 59.5% | 72.2% |
| Chinese(III) vs. Healthy+ Cirrhotic | AUC | 0.878 | 0.924 | 0.962 | 0.927 | 0.901 | 0.967 | 0.905 | 0.912 | 0.964 |
|  | Specificity | 98.7% | 92.5% | 93.4% | 99.6% | 96.6% | 96.6% | 99.1% | 95.0% | 94.3% |
|  | Sensitivity | 42.9% | 74.3% | 88.6% | 53.3% | 73.3% | 86.7% | 48.8% | 72.5% | 87.5% |
| Chinese(III) vs. Healthy | AUC | 0.899 | 0.956 | 0.985 | 0.937 | 0.926 | 0.980 | 0.919 | 0.940 | 0.982 |
|  | Specificity | 100.0% | 92.6% | 93.3% | 100.0% | 93.4% | 94.7% | 100.0% | 93.0% | 93.4% |
|  | Sensitivity | 42.9% | 82.9% | 94.3% | 53.3% | 77.8% | 88.9% | 48.8% | 80.0% | 91.3% |
| Chinese(III) vs. Cirrhotic | AUC | 0.848 | 0.877 | 0.928 | 0.908 | 0.856 | 0.943 | 0.881 | 0.865 | 0.934 |
|  | Specificity | 96.7% | 84.8% | 84.8% | 98.8% | 93.8% | 93.8% | 97.7% | 90.2% | 89.6% |
|  | Sensitivity | 42.9% | 74.3% | 88.6% | 53.3% | 73.3% | 86.7% | 48.8% | 72.5% | 86.3% |

**^a^**The diagnostic cutoff value of AFP was 400 ng/mL.
